# Global research on 24-hour movement behaviours guidelines in children and adolescents: a systematic review

**DOI:** 10.1186/s12966-025-01809-5

**Published:** 2025-08-08

**Authors:** Mosharop Hossian, Gregore Iven Mielke, Mehwish Nisar, Mark S. Tremblay, Asaduzzaman Khan

**Affiliations:** 1 School of Health and Rehabilitation Sciences, The University of Queensland, Brisbane, Qld 4072 Australia; 2 School of Public Health, The University of Queensland, Brisbane, Australia; 3 Children’s Hospital of Eastern Ontario Research Institute, 401 Smyth Road, Ottawa, ON K1H 8L1 Canada; 4 Department of Pediatrics, University of Ottawa, 401 Smyth Road, Ottawa, ON K1H 8L1 Canada

**Keywords:** Movement behaviours, Sedentary time, Physical activity, Adolescent health, Academic performance

## Abstract

**Background:**

Compliance with 24-hour movement behaviours (24-h MB) guidelines, which encompass moderate-to-vigorous physical activity (MVPA), recreational screen time (ST), and sleep, is associated with various health and developmental outcomes in children and adolescents. Despite growing research interest, a comprehensive synthesis of global research focusing on school-aged youth (5–17 years) is lacking. This systematic review mapped global research on 24-h MB guidelines in youth aged 5–17 years, charted publication trends, geographical spread, and summarised reported outcomes to inform research priorities.

**Methods:**

A systematic search (June 2016-July 2024) across six databases (PubMed, Scopus, Web of Science, SPORTDiscus, APA PsycInfo, Embase) identified 32,832 articles. Overall, 148 articles from 32 countries met inclusion criteria. Extracted data covered publication details, movement behaviours measures, article focus, and headline conclusion. ‘Compliance’ was defined as simultaneous adherence to all 24-h MB guidelines. Guided by the Behavioural Epidemiology Framework, articles were classified as prevalence, health and well-being, correlates, academic performance, intervention focused. Article quality was assessed with National Institute of Health tools.

**Results:**

Global research on 24-h MB guidelines has grown rapidly since 2016 but remains methodologically modest, with 68% articles (*n* = 132) originated on six high- or upper-middle-income countries. Most articles were cross-sectional (*n* = 128, 87%) and investigated prevalence (*n* = 141, 95%) or health and well-being (*n* = 79, 53%), followed by correlates (*n* = 40, 27%), academic performance (*n* = 8, 5%), and interventions (*n* = 3, 2%). Only 3% of observational and no intervention articles were rated high quality. Globally, compliance rates with 24-h MB guidelines were low (0–53.6%), with 87% (*n* = 122) articles reporting below 10%. Compliance with 24-h MB guidelines was associated with lower likelihood of obesity, mental health and cardiometabolic problems, and higher physical fitness, academic performance, and cognitive function. Correlates of 24-h MB guidelines compliance included age, gender, weight status, socioeconomic status, environmental pollution, parental support, and in-person schooling. Interventions promoting 24-h MB guidelines showed promising outcomes.

**Conclusions:**

Current research on 24-h MB guidelines is geographically skewed, with only 7% of articles on low- and middle-income countries data. Most evidence was cross-sectional, and no article achieved high methodological quality Future research should focus on under-represented regions, use longitudinal and experimental designs, and assess key outcomes such as academic performance to inform policy and practice for improving youth health and well-being globally.

**Supplementary Information:**

The online version contains supplementary material available at 10.1186/s12966-025-01809-5.

## Background

Daily movement behaviours (MB) are crucial for health and development of children and adolescents. An integrated approach is required to understand their relationships with health and development [1–3]. Recent research has emphasised the concept of “24-hour movement behaviours (24-h MB) guidelines,” encompassing sleep, moderate-to-vigorous physical activity (MVPA), and recreational screen time (ST) [1, 4, 5]. The interconnectedness of these three behaviours, as highlighted by Systems Theory, suggests that how they function within a single time-constrained system, where modifications in one component may produce impacts on the others [6]. For example, insufficient sleep may reduce energy for MVPA and increase ST, while MVPA might lead to better sleep [7]. This highlights why understanding these three behaviours together offers a comprehensive view of a child or adolescent’s daily MB. Fostering MB during childhood and adolescence can lead to improved physical and mental health, setting the stage for lifelong well-being [8–10]. The ages of 5–17, which encompass the typical school years [11, 12], are crucial for establishing lasting habits [13], as behaviours established at this stage can create enduring changes [8–10], unlike toddlers or preschoolers who are still developing basic routines and may not have the same consistency in their daily activities [14].

The 2016 Canadian 24-h MB guidelines for children and adolescents were the first to address the full range of daily movement behaviours, highlighting their importance for long-term health and development and setting a benchmark for public health initiatives globally [5]. The guidelines recommend that children (5–12 years) get 9–11 h of sleep per night, and adolescents (13–17 years) get 8–10 h, with both groups advised to engage in at least 60 min of MVPA daily and limit recreational ST to no more than 2 h per day [5, 15]. Compliance with these guidelines is crucial [16], as non-compliance may increases the risk of non-communicable diseases like obesity, cardiovascular disease, and mental health disorders [17–20]. Non-compliance may also affect academic performance, further highlighting the need for interventions to promote healthy MB [21–23]. However, a substantial proportion of children and adolescents worldwide do not comply with the recommended guidelines for 24-h MB [16], resulting in concerns about possible health risks and poor academic performance [24, 25].

Variations in the research focus across different regions have led to an incomplete and fragmented understanding of global research using 24-h MB guidelines. Reviews have often emphasised certain aspects of these behaviours depending on regional research priorities, leading to gaps in comprehensive knowledge. For example, while some regions may concentrate heavily on specific movement patterns, others may overlook them entirely, creating a fragmented view of the global landscape. Europe, for instance, has the highest number of published studies on physical activity (PA), while countries like the United Kingdom (UK), Australia, Canada, and the United States of America (USA) have the most published papers on sedentary behaviours (SB) and sleep, highlighting potential research gaps in other regions [26–28]. However, to develop a holistic understanding, it is essential to synthesise evidence that examines compliance with all three 24-h MB guidelines, rather than focusing on single or paired behaviours.

Prior reviews on 24-h MB guidelines have often focused on isolated outcomes associated with 24-h MB, such as physical health indicators [3, 24], cognitive outcomes [3, 24, 29], and psychosocial development [29], without integrating the overall perspective of 24-h MB guidelines into a cohesive concept. Consequently, gaps remain in mapping overall research on 24-h MB guidelines, especially among school-going children and adolescents. Given the critical role of 24-h MB guidelines in physical [10, 30–35], cognitive [35–39], and psychosocial [40, 41] development during childhood and adolescence, a structured synthesis of global research is needed to map that can inform research direction of the field.

Guided by gaps that remain in the existing literature, this systematic review had three primary objectives: (1) to map the distribution of research on 24-h MB guidelines among children and adolescents (5–17 years), across the foci of the articles; (2) to chart the global growth and geographical spread of this literature since 2016; and (3) to summarise core study characteristics (design, sample, measurement) and their variation across articles. As a secondary aim, the review presents a narrative synthesis of reported findings to provide an overview of current evidence and identify directions for future research.

## Methods

### Study design

A systematic search of global research using 24-h MB guidelines from June 2016 to July 2024 was conducted, followed by data extraction and analysis This time frame was chosen because the world’s first 24-h MB guidelines for children and adolescents were introduced in 2016, and the current review specifically targeted articles that addressed all three 24-h MB guidelines together. Recent reviews were reviewed to identify additional articles. This systematic review followed the PRISMA guidelines [42] and was registered with PROSPERO (CRD42023430572) [43]. Covidence was used for efficient reference management [44].

### Search terms and strategies

A systematic search was conducted across PubMed, Scopus, Web of Science, SPORTDiscus, APA PsycInfo, and Embase using search terms grouped into MB, guidelines, and age. The search strategy was adapted for each database. Full search strategy details are provided in the Supplementary Search Strategy File.

### Inclusion and exclusion criteria

The review included articles on generally healthy children and adolescents aged 5–17 that followed 24-h MB guidelines, covering MVPA, recreational ST, and sleep. All study designs were accepted except for systematic reviews, meta-analyses, and protocols. Only English-language peer-reviewed articles were included, while articles on clinical populations, adults, infants, and grey literature were excluded.

### Study selection, quality assessment, and data extraction process

Two reviewers (MH and MN) separately assessed the articles for inclusion by first reviewing titles and abstracts, followed by full-text review. Disagreements were resolved by a third reviewer (AK or GIM). Methodological quality of the articles was assessed using the Quality Assessment Tool developed by National Institute of Health [45]. Data were then extracted using a standardised template, covering details like author(s), publication year, data type, country, journal name, study participants, study design, MVPA, ST, and sleep duration measures, article focus and headline conclusion. For articles involving multiple countries, research output was attributed to all contributing nations. Unless otherwise specified, the terms ‘compliance’ or ‘adherence’ refer to meeting all three 24-h MB guidelines simultaneously.

### Classification of extracted data

A dual classification system, based on the country’s economic status and research foci, was used to better understand research productivity using 24-h MB guidelines. By dividing countries by economic status using World Bank classifications (High-income, Upper-middle-income, Lower-middle-income, and Low-income) [46], we can better examine how financial resources may impact research output [47], a more specific measure than broader indices such as the Human Development Index (HDI) [48]. Research foci categorisation were guided by the adopted version of BEF [49], covering prevalence, correlates, health and well-being, academic performance, and interventions, helps identify key trends and gaps. To systematically map and organise the existing literature on 24-h MB guidelines in children and adolescents, the Behavioural Epidemiology Framework (BEF) was adopted as an organising framework [49]. The BEF provides a structured continuum for behavioural research across five sequential phases: (i) establishing links between behaviours and health, (ii) measuring behaviours, (iii) identifying correlates, (iv) developing interventions, and (v) translating research into practice. BEF has been extensively applied in MB research [50–52] and offers a logical structure to examine the focus and maturity of existing studies. In this review, articles were categorised according to the first four BEF phases. Phase 1 included articles linking 24-h MB guidelines to physical, mental, and cognitive health outcomes; Phase 2 comprised articles reporting measurement and prevalence of 24-h MB guidelines compliance; Phase 3 included articles on correlates of adherence to 24-h MB guidelines; and Phase 4 captured intervention-based articles aiming to improve compliance with 24-h MB guidelines. Academic performance outcomes were also included as a distinct focus, aligning with BEF’s emphasis on developmental outcomes given their critical importance in school-aged children and adolescents [49]. Phase 5 (“Translation into practice”) was not included, as the present review focused on synthesising peer-reviewed literature rather than evaluating implementation or policy translation. Articles were classified as prevalence focused when they reported overall rates of guideline compliance and/or descriptive subgroup comparisons (e.g., by gender or age). Articles that examined relationships with adherence using any inferential method, including regression models, correlation coefficients, χ² tests, or decision-tree analysis or similar, were considered as correlates focused; for these we extracted the significant associations exactly as reported, without evaluating the specific analytic technique.

### Synthesis of results

R software was used to summarise the distribution of research using 24-h MB guidelines on children and adolescents (5–17 years). A foci-based bar chart showed yearly trends, while a global map highlighted the geographical distribution of research articles. Tables were organised according to focus of the articles to show article characteristics, providing a clear overview of the research landscape. In addition, reported findings within each focus were narratively synthesised to highlight recurrent associations, emerging themes, and evidence gaps.

## Results

**Fig. 1:**
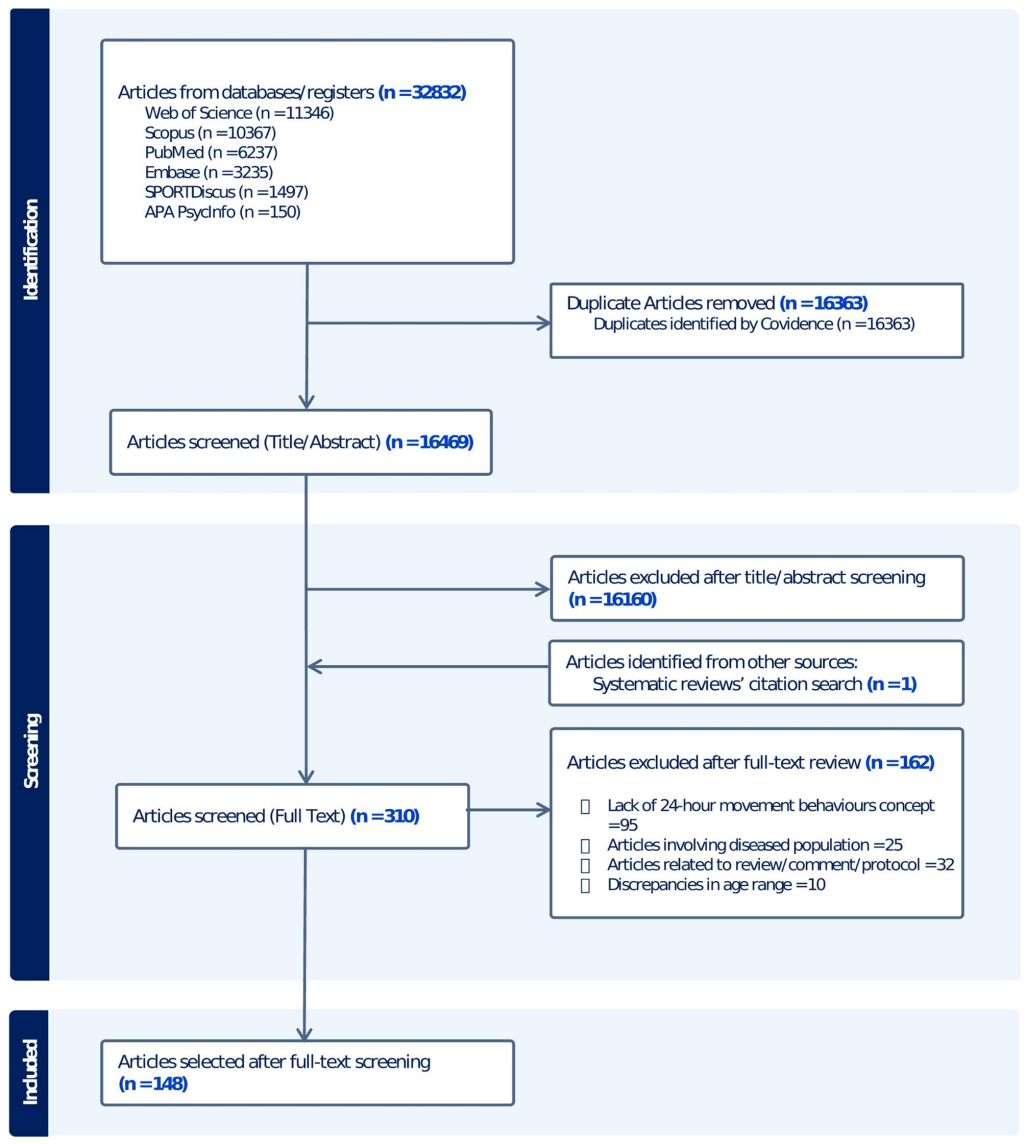
PRISMA flow diagram for articles selected for the systematic review

Of the initial 32,832 articles identified, 16,469 remained after removing duplicates. Following title/abstract screening and full-text reviews for eligibility criteria, 147 articles were included. This selection was further supplemented by one article from previous reviews. The process flow for article selection is illustrated in Fig. 1.

### Quality assessment

The results of the methodological quality assessment are provided in the supplementary quality assessment file. Only 3% (*n* = 5) of the observational articles (cross-sectional or longitudinal designs) were rated as high-quality, while 97% (*n* = 140) were rated as fair quality. None of the intervention articles (*n* = 3) achieved a high-quality rating, with only one deemed fair quality.

### Publication overview

**Fig. 2:**
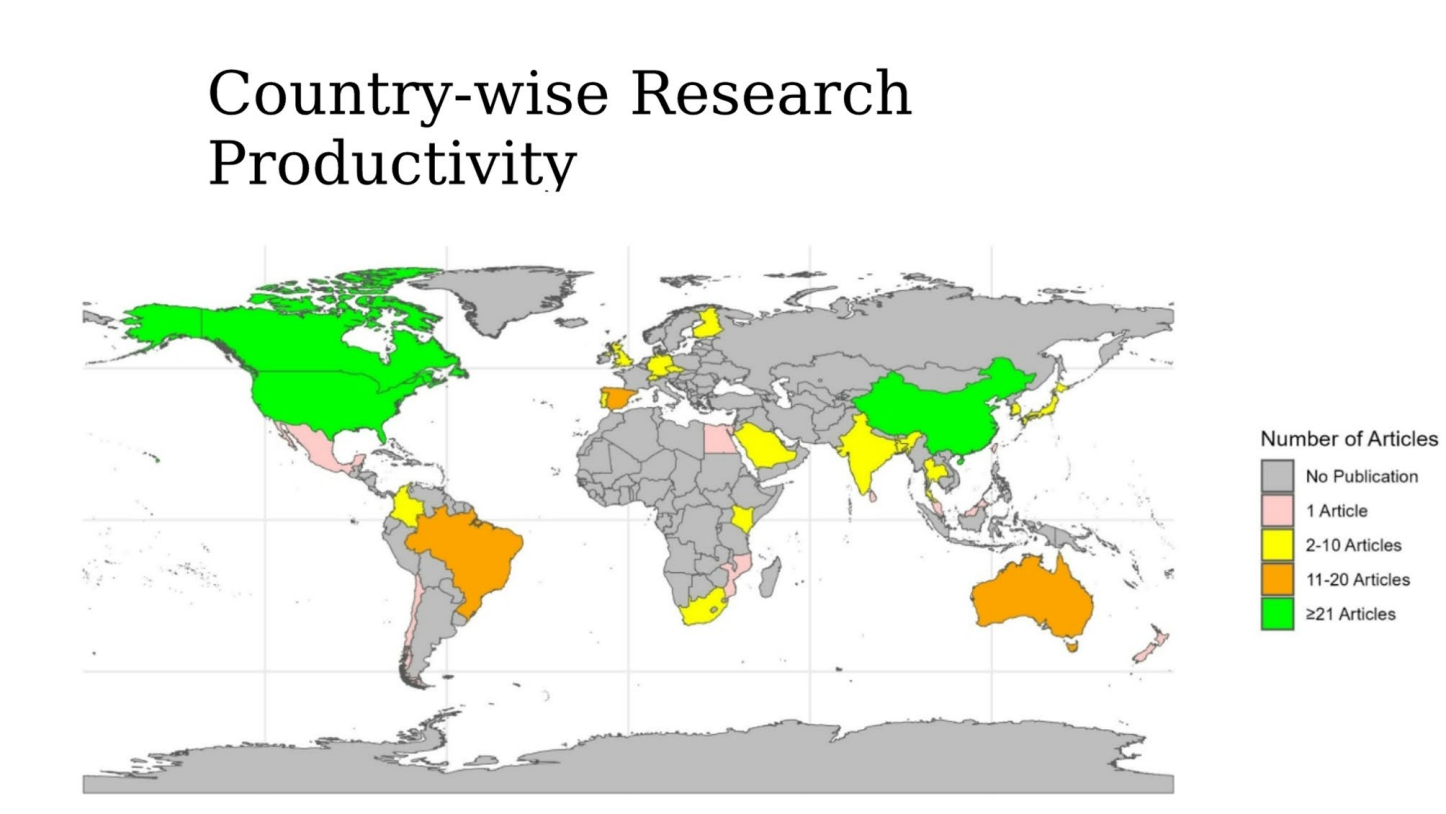
Country-wise research productivity in research using 24-hour movement behaviours guidelines

Articles using 24-h MB guidelines were found on 32 different countries. The number of articles varied on country to country, ranging from 1 to 33, with six countries contributing two-thirds of the articles (Fig. 2).

China led in the research using 24-h MB guidelines, producing 33 articles, accounting for 22% of the total. This was followed by Canada and USA, contributing 30 (20%) articles each. Spain, Australia, and Brazil also made notable contributions, with 16 (11%), 12 (8%), and 11 articles (7%), respectively. Other major contributors included Japan, the UK, Finland, Kenya, Portugal, India, and Republic of Korea. It was evident that a few countries, especially from the high and upper-middle income brackets, were leading the way, whereas contributions from low- and middle-income countries (LMIC) remain limited, only 7% (Supplementary Table 1).

### Movement behaviours research foci

**Fig. 3:**
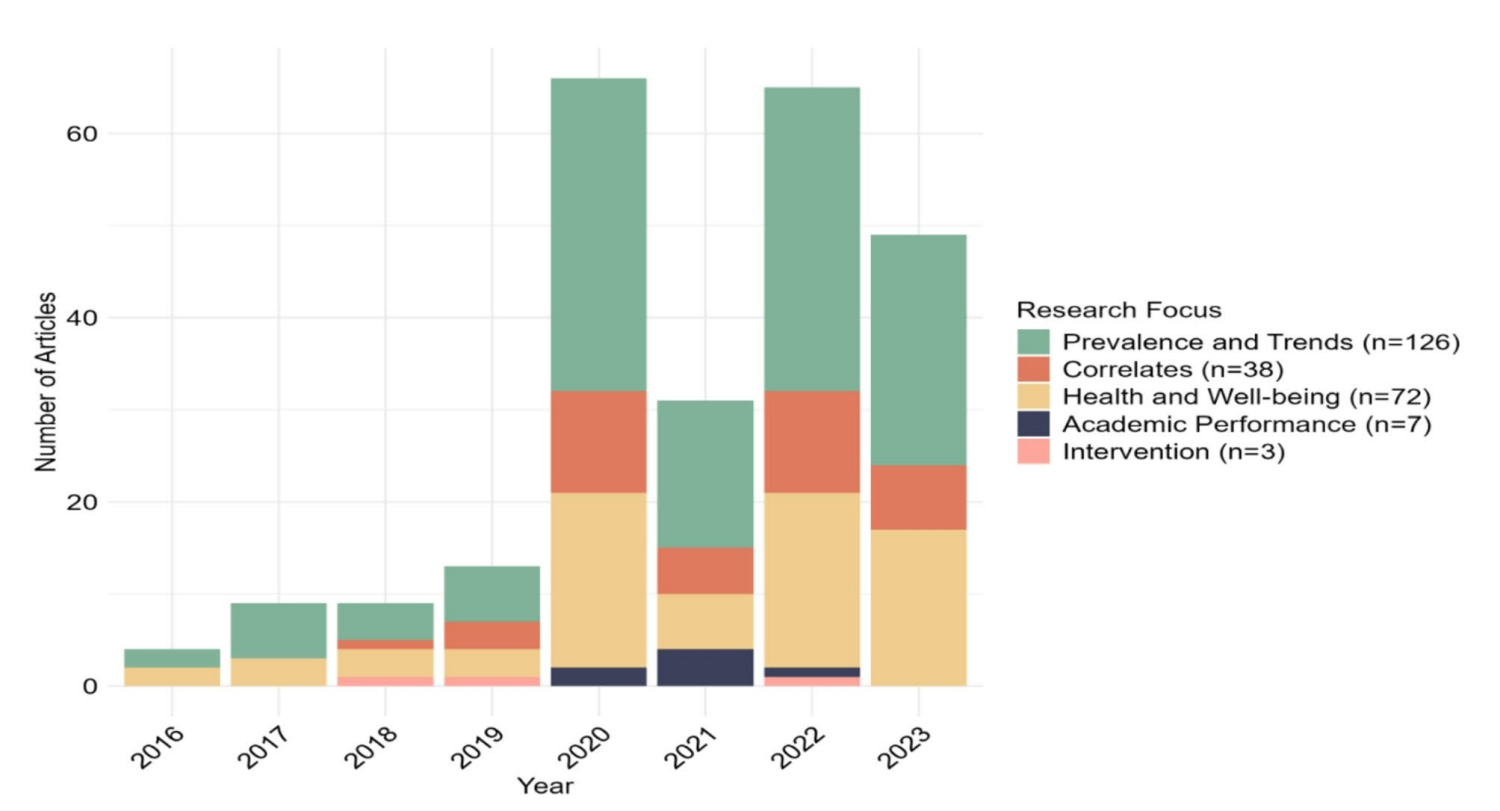
Number of articles using 24-hour movement behaviours guidelines published by research foci 2016–2023

Over the years, there was a steady growth in articles across almost all identified research focuses, although a noticeable dip in the number of articles published occurred in 2021 (Fig. 3).

(***Note***: Articles from 2024 are excluded due to the year being only partially covered in the search).

### Prevalence and trends research

A majority of all included articles (95%, *n* = 141) investigated prevalence of adherence with 24-h MB guidelines, predominantly using a cross-sectional design (89%, *n* = 125), with fewer employing a longitudinal design (13%, *n* = 18), and a quasi-experimental design (1%, *n* = 1) (Supplementary Table 2). Only four articles assessed time trends in 24-h MB guidelines compliance. A small proportion of articles used device-measured data (MVPA 18%, *n* = 25; recreational ST: 1%, *n* = 1; sleep duration: 10%, *n* = 14), with a few articles used both self-reported and device-measured data together. Sample sizes ranged from 49 to 322,965 participants, with 34% (*n* = 48) articles used nationally representative datasets. Globally, adherence to the 24-h MB guidelines is exceptionally low [8–10, 17–23, 30–41, 53–171], ranging from 0 to 53.6%, with 87% (*n* = 122) articles reported less than 10% compliance, with some articles reporting compliance rates below 1% [18, 33, 34, 68, 71, 74, 76, 84, 95, 98, 109, 111, 141, 156, 163, 171] (Supplementary Table 3). Moreover, 26% (*n* = 36) articles reported over 25% of children and adolescents did not comply with any 24-h MB guideline, with some articles reporting up to 66% of participants failed to meet any [9, 18, 23, 34–37, 39, 56, 72–74, 83, 84, 87, 91–95, 97, 101, 107, 109, 111, 118, 126, 127, 136, 137, 141, 144, 145, 151, 156, 160]. A gender difference was reported in 24 articles, with 18 articles finding that boys had a higher prevalence of adherence than girls [18, 20, 38, 54, 59, 63, 91, 93, 102, 103, 114, 124, 127, 131, 133, 148, 158, 168], while four articles reported the opposite, with girls demonstrating higher prevalence of adherence than boys [68, 71, 120, 125]. Furthermore, two articles reported mixed findings among children and adolescents [78, 106]. In general, older aged participants showed lower prevalence of adherence compared to younger aged ones [20, 33, 35, 40, 54, 65, 72, 78, 82, 85, 93, 96, 102, 106, 113, 115, 121, 122, 133], though, two articles reported the opposite, with younger children showing lower prevalence of adherence than older ones [118, 167]. A greater proportion of healthy weight children met al.l three guidelines concurrently compared to those who were thin, overweight, obese, or morbidly obese [131]. Rural children typically showed higher prevalence of adherence than urban children [64, 116, 163], while prevalence of adherence declined during the transition from primary to secondary school [170]. Environmental factors also played a role, with higher prevalence of adherence observed in areas with lower air pollution [128], and lower prevalence of adherence among children exposed to environmental tobacco smoke compared to those not exposed [154]. Prevalence of adherence was also higher on weekends than weekdays [84, 170], greater during in-person schooling than virtual learning [117], and increased after school reopening compared to closures [164]. Self-reported data tend to overestimate compliance compared to accelerometer-measured data, with significant differences [21, 74, 95].

### Correlates research

Approximately 27% (*n* = 40) of the total 148 articles focused on assessing correlates of compliance with all three 24-h MB guidelines (Supplementary Table 2), with none assessing its determinants. Of these 40 articles, most (93%, *n* = 37) used cross-sectional design, while only four articles used longitudinal design. A small fraction used device-measured data (MVPA: 20%, *n* = 8; recreational ST: 3%, *n* = 1; sleep duration: 18%, *n* = 7), with a few articles used both self-reported and device-measured data together. Sample sizes ranged from 485 to 322,965 participants and 23 articles (*n* = 58%) were based on nationally representative datasets. Several demographic, socioeconomic, environmental, parental, and school-related factors were associated with adherence to 24-h MB guidelines (Supplementary Table 4). Demographic factors included age, gender, race/ethnicity, and BMI, with older children and adolescents less likely to meet the guidelines compared to younger ones [19, 79, 80, 113, 116, 129, 145, 165]. Males were generally more likely than females to adhere [84, 85, 113, 114, 145], though one article reported that females were 1.5 times more likely than males to meet al.l recommendations [162]. Black, Asian, and other racial groups were less likely to meet the guidelines compared to non-Hispanic/white peers [63, 114, 132, 162]. Overweight, obese, and underweight children were less likely to adhere compared to those with a healthy weight [62, 63, 82, 113, 131, 162]. Socioeconomic factors such as household income, parental education, and community characteristics were also relevant. Multiple articles reported that children and adolescents from families with higher socioeconomic status were more likely to meet the guidelines [19, 62, 113, 129] and those from the most deprived communities were less likely to meet 24-h MB guidelines [145], although some other articles reported children and adolescents from lower-income families [120], lower-middle-income areas [162] and less developed regions [114] were more likely to meet the guidelines. Environmental factors included urban vs. rural residence, air pollution, and outdoor opportunities. Children and adolescents living in metro areas [60] and attending urban schools [64] were less likely to adhere, though another article reported that urban children were more likely to adhere [80]. Children who spent more time outdoors were less likely to meet the guidelines [64], whereas greater outdoor opportunities, such as access to parks [77], walking or biking [79], and participation in community sports [70], were linked to higher likelihood of adherence, with changes in outdoor time showing a strong association with adherence [134]. Environmental exposures such as high air pollution [128] and exposure to environmental tobacco smoke [154] were linked to lower likelihood of adherence. Parental and family factors included support, encouragement, engagement in physical activity, screen time control, family structure, parental education, family history of chronic disease. Children with older parents were more likely to meet al.l guidelines [120, 130]. Findings on parental education were mixed, with some articles reported children and adolescents with higher parental education were less likely to adhere [64, 120, 162], while some other articles reported that children and adolescents with higher parental education were more likely to meet al.l three guidelines [19, 130, 132]. Boys with a family history of type 2 diabetes were less likely to meet the guidelines [59]. Parental encouragement [78], support [78, 106], engagement in physical activity [78], and family dog ownership [78] were positively linked to adherence. Parental control played a role, with children whose parents strongly believed they could restrict screen time being more likely to meet the guidelines [79, 134]. The use of online resources to support movement behaviours was also positively linked to adherence [134]. Knowing where to go for help was associated with a higher likelihood of meeting the recommendations [104]. Family dynamics were also linked to adherence, with children from families with high acceptance, high monitoring, and low conflict being more likely to meet the guidelines than those from families with lower acceptance, lower monitoring, and higher conflict [172]. Families with multiple children in the same age-based sleep guideline category were more likely to adhere than those with children in different categories [138]. School-related factors included school type, physical education participation, screen time habits, and academic performance. Attending in-person school was linked to a higher likelihood of meeting the guidelines compared to virtual schooling [117]. A safe school environment was positively associated with adherence, particularly among females [104]. Gymnasium school type and better subjective school performance were also linked to higher likelihood of adherence [113]. Similarly, children attending Islamic schools were twice as likely to meet al.l three recommendations compared to those in secular schools [146]. Attending school in the afternoon was associated with a greater likelihood of meeting all three guidelines [114]. Students who avoided screen time before school were more likely to adhere [116]. Participation in daily physical education lessons increased adherence, with each extra hour per week of participation further improving the likelihood of meeting all three recommendations [149]. Behavioural factors included diet quality, swimming ability, and psychological status. Children with better diet quality [129] and those who could swim [116] were more likely to meet the guidelines. Theory of Planned Behaviour constructs were linked to adherence, with habit strength having association with adherence [173], while past behaviour showed a significant association with adherence [96, 173]. Children and adolescents with Attention-deficit/hyperactivity disorder (ADHD) [113], depressive symptoms [62, 63, 113], or substance use [63, 113] were less likely to meet al.l three guidelines.

### Health and well-being research

About 53% (*n* = 79) of the total 148 articles focused on the relationship of compliance with 24-h MB guidelines with well-being and/or health-related indicators or outcomes (Supplementary Table 2). Out of these 79 articles, the vast majority, 87% (*n* = 69) employed a cross-sectional design, whereas 16% (*n* = 13) used a longitudinal design. A small number of articles collected data using devices (MVPA: 18%, *n* = 14; recreational ST: 0%, *n* = 0; sleep duration: 9%, *n* = 7). The sample size used in these articles varied significantly from 185 to 238,440 participants, and 28% (*n* = 22) of the articles utilised nationally representative datasets. No articles with longitudinal or intervention design established causal effect of compliance with 24-h MB guidelines on well-being or health-related outcomes. Children and adolescents who meet al.l three 24-h MB guidelines were consistently associated with beneficial health outcomes across multiple domains (Supplementary Table 5). Adherence to these guidelines was linked with higher health-related quality of life (HRQoL), better self-rated physical, mental, and psychosocial health, and increased life satisfaction compared to those meeting fewer recommendations [32, 40, 53, 61, 67, 75, 83, 110, 115, 118, 136, 137, 141, 174]. A clear dose-response relationship exists, whereby adherence to higher number of 24-h MB guidelines corresponds to improved psychosocial health, reduced mental comorbidity and life stress, and increased self-esteem [41, 127, 136, 137, 155]. Mental health benefits were particularly significant, with adherence linked to lower risks of anxiety, depression, emotional problems, suicidal ideation, and suicide attempts, while non-adherence is associated with elevated sadness, loneliness, and stress [8, 18, 32, 35, 41, 61, 65, 68, 88, 92, 93, 103, 110, 124, 129, 140, 151, 153, 157, 160]. Children who adhered to the guidelines were also less likely to require mental health visits [175] and had reduced risk of engaging in problem behaviours, including internalising and externalising difficulties, peer problems, conduct problems, hyperactivity [41, 94] and more likely to have better strengths and difficulties score [31]. Cognitive benefits were similarly evident, with adherence linked to improved global cognition, cognitive flexibility, and executive functioning, whereas non-adherence was related to poorer cognitive performance, notably in shifting efficiency and rule discovery [35–39]. Additionally, non-adherence was also associated with poorer executive functioning, including difficulties in shifting efficiency and rule discovery [38]. Furthermore, children and adolescents who did not meet the guidelines had higher odds of Internet addiction [126] and were more likely to engage in substance use, including cannabis consumption [102]. Social outcomes were also positively associated, as those adhering to all guidelines were less likely to experience bullying, whether as victims or perpetrators, with stronger associations observed in cases of cyberbullying and verbal bullying [91, 176]. Meeting all three guidelines was also associated with reduced physical and mental pain, fatigue, irritability, and lethargy [164]. Adherence to all three guidelines is linked with a lower likelihood of obesity and overweight among children and adolescents [10, 19, 20, 30, 56, 69, 81, 86, 105, 119, 130, 159]. Adherent children and adolescents were more likely to have lower body mass index (BMI), reduced body fat, smaller waist circumference, and lower overall adiposity [10, 30–35]. Meeting these guidelines was also associated with better cardiometabolic health, including lower blood pressure, insulin resistance, triglyceride levels, and improved lipid profiles [9, 17, 31, 148]. Furthermore, adherence was also associated with greater aerobic fitness, cardiorespiratory fitness, muscular fitness, muscular strength, and overall physical fitness [31, 111, 142, 161]. Although adherence did not appear to significantly associated with measures such as grip strength, sit-ups, sit-and-reach, or performance in the 20-meter shuttle run [89], it was associated with better performance in the standing long jump [142]. Adherence was further associated with better self-rated physical health, fewer physical comorbidities [101, 155]. A lower likelihood of type 2 diabetes [9], myopia [152], and abdominal obesity in later life [10], as well as higher cortical grey matter volume [35] were evident among children and adolescents meeting the guidelines. Longitudinal studies supported that adherence with 24-h MB guidelines were associated with the higher likelihood of better cognitive [35], behavioural [8, 35], physical health [35, 115], HRQoL [75], life satisfaction [115]; lower likelihood of adiposity levels [33], anxiety and depressive symptoms [140], cardiometabolic risk [96], overweight, obese [10], and having type 2 diabetes mellitus [9]. Adherence to all three 24-h MB guidelines was also associated with healthier dietary patterns. Children and adolescents meeting these guidelines were more likely to adhere with Mediterranean diet and consuming fruits, vegetables, fish, cereals, or grains regularly, while being less likely to consume commercially baked goods or sweets [58, 108]. Conversely, non-adherence was associated with dietary patterns characterised by lower fibre intake, reduced consumption of vitamins B6, B12, C, selenium, and magnesium, and higher intake of saturated fatty acids [69].

### Academic performance research

Eight articles investigated the relationship between compliance with 24-h MB guidelines and academic performance, of which seven articles were cross-sectional (Supplementary Table 2). A minority of articles used both self-reported and device-measured data together (MVPA: *n* = 1; recreational ST: *n* = 0; sleep duration: *n* = 1). Most articles were on Australia (*n* = 2) and Spain (*n* = 2), with sample ranging from 501 to 67,281. None of the articles assessed causal effects of adherence with 24-h MB guidelines on academic performance. Adherence to all three guidelines was associated with improved academic performance across various subjects, including mathematics, English, and Chinese, compared to meeting fewer or no guidelines [22, 23, 99, 144] (Supplementary Table 6). Adherence with a greater number of 24-h MB guidelines correlated progressively with better numeracy and overall academic achievement, particularly notable in primary and middle school students [21–23, 97, 99, 107, 144]. Adolescents meeting all guidelines showed significantly greater odds of reporting better academic performance, especially evident among students in primary and junior middle schools, but not in higher grades [107, 144].

### Intervention research

Three articles reported interventions assessment, with two using quasi-experimental and one experimental design (Supplementary Table 2). One article utilised device-measured data for MVPA, while one article used both self-reported and device-measured data to assess recreational ST. The articles predominantly were on Spain (*n* = 2), had participant numbers ranging from 121 to 210. Interventions aimed at improving adherence to 24-h MB guidelines have shown promising outcomes (Supplementary Table 7). One experimental school-based intervention reported improvements in students meeting the guidelines compared to control students and their own baseline measures, with somewhat stronger effects observed in boys [177]. Another intervention indicated that parents who read integrated guidelines appeared more likely to apply the recommendations, particularly when they understood the messages clearly [178]. All three identified interventions were school-aged, multicomponent efforts that addressed the 24-h MB guidelines, yet they differed markedly in delivery strategy. Paths of the Pyrenees embedded health-literacy and behaviour-change activities across the curriculum and school community [177]; the Canadian randomised experiment tested whether presentation of the guidelines (integrated vs. segregated) improved parents’ knowledge and action planning [178]; and the Spanish Creating Active Schools (CAS) + Self-Determination Theory (SDT) programme used weekly tutorial sessions to build skills and motivation for balancing 24-h MB guidelines [125]. The article [125] reported each behaviour compliance separately because few individuals met al.l three guidelines together and reported a significant within-group reduction in weekday screen-time but no intervention-versus-control difference, likely owing to already-healthy baselines in the control class.

## Discussion

This comprehensive review of global research using 24-h MB guidelines in children and adolescents (aged 5–17 years) reveals key trends and gaps. Research volume surged from two articles in 2016 to peaks of thirty-four in 2020 and thirty-three in 2022, although 68% of articles focused on only six countries, with a mere 7% addressing LMIC. Adherence to 24-h MB guidelines remained critically low worldwide, with some regions showed below 1% compliance, and up to 66% of children failing to meet any guidelines. Device-based measurements were underutilised, whereas cross-sectional designs dominated the literature. Most articles (95%) reported prevalence and trends, with a limited focus on academic performance and interventions. Only 27% of the articles assessed factors associated with 24-h MB guidelines compliance including age, gender, weight status, socioeconomic status, environmental pollution, parental support, and in-person schooling. No articles reported the determinants of guideline adherence, revealing a gap in causal understanding. Health and well-being outcomes were addressed in 53% of the articles, compliance with 24-h MB guidelines were associated with lower likelihood of obesity, mental health problems, and adverse cardiometabolic profiles, as well as higher likelihood of better physical fitness, and cognitive functioning. However, causal effects of adherence with 24-h MB guidelines on health and developmental outcomes remain unexplored owing to the prevalence of cross-sectional designs, although longitudinal studies suggested long-term benefits. Academic performance has scarcely been examined, with only eight articles. Adherence with 24-h MB guidelines correlated with academic achievement, however, no causal effect of adherence with 24-h MB guidelines on academic performance has been established. Intervention research were limited to three articles, with two interventions (a school-based program with stronger effects in boys and a parent-targeted strategy) reporting improvements in prevalence of adherence with 24-h MB guidelines. Overall, future research should prioritise underexplored areas, such as academic performance and interventions, employ longitudinal and experimental designs, utilise more device-based measures, and expand geographically, especially on LMIC, to improve adherence and promote health and academic performance.

The findings of the review show consistent global growth in 24-h MB research, with most articles conducted using data from high and upper-middle income countries such as China, Canada, the USA, Spain, Australia, and Brazil. For instance, Canada’s 2016 introduction of the 24-h MB positioned it as a pioneer [5]. Only 7% of the articles were on LMIC. This distribution of articles using 24-h MB guidelines mirrored broader trends in the PA, SB, and sleep literature, where research is predominantly driven by high-income contexts [26–28]. Factors such as differences in governmental research priorities, funding availability, and methodological capacity in resource-poor settings may contribute to this imbalance. Although the field is evolving rapidly, with the number of published articles rising from two in 2016 to 34 in 2020 and 33 in 2022, and the incorporation of diverse methodologies focus and regions, the current evidence base remains skewed. To establish a more inclusive global evidence base, future research should focus on expanding studies in LMIC by adapting methodologies to local contexts and aligning research agendas with regional public health needs.

A persistently low prevalence of adherence to the 24-h MB guidelines was observed in this review, though prevalence of compliance often varied by gender, age group, and residential setting As children progress into adolescence, increased academic demands and autonomy may lead to a decline in MVPA [179], increase in recreational ST [180], and disrupted sleep [181], all of which may reduce overall compliance [20, 33, 35, 40, 54, 65, 72, 78, 82, 85, 93, 96, 102, 106, 113, 115, 121, 122, 133]. Boys tend to show higher prevalence of adherence with 24-h MB guidelines than girls [18, 20, 38, 54, 59, 63, 91, 93, 102, 103, 114, 124, 127, 131, 133, 148, 158, 168], possibly because of societal norms [182]. These societal preferences often lead to boys having more opportunities for MVPA and participating less in recreational ST [183]. Moreover, participation in active pursuits promotes structured routines that may support consistent sleep patterns [184]. Rural children often showed higher prevalence of adherence with 24-h MB guidelines than their urban peers [64, 116, 163], possibly due to greater access to outdoor spaces [185, 186], fewer screen time distractions [187] and more consistent sleep environments [188, 189]. Although some studies reported no difference [190] or even worse [191] MVPA levels in rural areas, with rural children also sometimes showing higher recreational ST [191] and poorer sleep quality [192]. Cultural and infrastructural differences also contribute to regional variation in adherence [16]. These findings suggest the need for multifaceted context-specific interventions to improve the prevalence of adherence with 24-h MB guidelines among diverse groups of children and adolescents worldwide.

The review revealed a heavy reliance on cross-sectional study designs in research using 24-h MB guidelines, limiting the ability to observe temporal changes or establish causality [193], which are both essential for effective interventions and guidelines development or adoption [194]. This reliance likely stems from the resource and funding limitations [193]. Notably, all longitudinal and experimental studies have been conducted in high-income countries. To address this, more robust designs, collaborative funding, capacity building, and partnerships are needed in LMIC.

In addition, Self-report measures are commonly used in research [37, 67–71, 75, 76, 80] using 24-h MB guidelines because of their cost-effectiveness [195–199], but they introduce biases, such as over-reporting and social desirability [197, 198, 200]. Device-based methods, such as accelerometry, offer more accuracy but are used less because of cost and technical demands [201]. Self-reports often showed higher adherence [21, 74], possibly because of recall errors. Efforts should focus on making advanced tools more accessible, fostering collaboration for equipment sharing, and increasing funding. In resource-limited settings, establishing reliable questionnaires is crucial for an accurate assessment of adherence with 24-h MB guidelines.

The included articles in this review indicated that 95% of the articles focused on the prevalence of meeting guidelines, and 53% explored relationships with health-related outcomes. These articles lay a solid foundation for understanding the key aspects of adherence with 24-h MB guidelines, providing benchmarks for measuring adherence and tracking progress. However, the current focus on prevalence and health often overlooks other areas, such as the relationship between adherence with 24-h MB guidelines and academic performance, or the effectiveness of interventions aimed at improving the prevalence of 24-h MB guidelines compliance. Early research using 24-h MB guidelines may have been driven by health promotion and disease prevention frameworks [49]. While health promotion and disease prevention remain crucial, the scope of research using 24-h MB guidelines has since evolved to include other domains, such as academic performance. Although most research remains concentrated on six countries, the field is gradually expanding to other regions. Expanding research across countries is crucial for understanding regional dynamics. Replicating studies in diverse contexts validates the findings, but future research must combine replication with innovative, context-specific studies to address unique sociocultural and environmental influences on adherence with 24-h MB guidelines.

Several demographic, socioeconomic, environmental, parental, and school-related factors were associated with adherence to 24-h MB guidelines. A higher socioeconomic status [19, 62, 113, 129] often provides more access to organised sports [202], recreational activities [203], and environments conducive to healthy sleep [204]. Although some other findings suggest that children and adolescents from lower-income families [120], lower-middle-income areas [162] and less developed regions [114] were more likely to adhere with 24-h MB guidelines, possibly due to different lifestyle priorities. Parental support [106, 172] and access to outdoor activities [77, 79] also play crucial roles in promoting MVPA [205, 206], reducing recreational ST [207, 208], and supporting better sleep [209, 210]. In contrast, depressive symptoms [62, 63, 113], or substance use [63, 113] and weight status [62, 63, 82, 113, 131, 162] were strongly associated with lower likelihood of adherence with 24-h MB guidelines, as they may reduce the motivation for MVPA [211, 212], increase recreational ST [213, 214], and disrupt sleep patterns [215, 216]. These findings underscore the complex interplay among mental health, lifestyle, and environmental factors. However, more research is needed to understand the causal mechanisms behind these correlations to create effective targeted interventions to improve the prevalence of adherence to the 24-h MB guidelines. At present, only three published articles addressed interventions to promote 24-h MB guidelines, leaving the intervention evidence base very small. In the meantime, evidence-mapping of combined compliance adds value: it highlights the demographic, social and environmental conditions that jointly support meeting all three recommendations and can steer the next generation of integrated programmes. Behaviour-specific correlates remain essential for fine-tuning individual components, but without first understanding the factors that foster overall 24-h MB guidelines compliance we lack a solid empirical footing for building or scaling multifaceted interventions.

Adherence to the 24-h MB guidelines were associated with lower likelihood of obesity [10, 19, 20, 30, 56, 69, 81, 86, 105, 119, 130, 159], mental health problems [8, 18, 32, 35, 41, 61, 65, 68, 88, 92, 93, 103, 110, 124, 129, 140, 151, 153, 157, 160], and adverse cardiometabolic profiles [9, 17, 31, 148], as well as higher likelihood of better physical fitness [31, 111, 142, 161], and cognitive functioning [35–39]. These potential outcomes likely stem from the interaction between MVPA, reduced recreational ST, and adequate sleep. MVPA may enhance cardiovascular and metabolic health by improving the circulation, heart function, and insulin sensitivity [217]. Similarly, reducing recreational ST could help maintain the blood flow and energy balance [218]. Adequate sleep may regulate key hormones, such as cortisol, leptin, and ghrelin, support stress management, control appetite, and facilitate recovery [219]. Together, adherence with these 24-h MB guidelines may create a synergistic effect that promotes efficient circulation, hormone regulation, and physiological balance. Additionally, they may contribute to improved cognitive function and emotional well-being through enhanced brain plasticity [220], mental clarity [221], and mood stabilisation [222]. Although longitudinal studies suggest lasting benefits such as improved cardiovascular health [96], mental well-being [115, 140], and cognitive function [35], further research is needed to establish causality and confirm how compliance with 24-h MB guidelines contribute to sustained health outcomes. In addition, experimental studies are needed to clarify the specific mechanisms and interactions of these behaviours, refine the guidelines, and optimise health strategies across diverse populations.

Understanding how adherence to 24-h MB guidelines are associated with academic performance is crucial, as it helps children and adolescents develop skills that are essential for learning, social interaction, and self-esteem [223]. Previous research have shown that adherence to all three 24-h MB guidelines was associated with improved academic performance across various subjects, including mathematics, English, and Chinese, compared to meeting fewer or no guidelines [22, 23, 99, 144]. Following a greater number of 24-h MB guidelines was more likely to be progressively associated with better numeracy and overall academic achievement, particularly notable in primary and middle school students [21–23, 97, 99, 107, 144]. These behaviours work together to support learning, memory retention, and attention. However, much of the current research focuses only on academic grades [21–23], while academic performance includes cognitive skills and attitudes, academic behaviours, and academic achievement [224]. Therefore, further research is needed to explore the full impact of adherence with 24-h MB guidelines on all facets of academic performance and investigate potential causal effects.

Interventions to improve the prevalence of meeting 24-h MB guideline focus on structure, engagement, and reinforcement. For example, School programs that incorporate MVPA during lessons and breaks effectively incorporate movement into students’ daily routines [177]. Parent-involved programs promoting a holistic approach to MVPA, sleep, and recreational ST may be more effective [178]. Future interventions should extend structured environments to home life, provide continuous support, and encourage family involvement. More research is needed to develop evidence-based interventions that promote lasting adherence to 24-h MB guidelines in children and adolescents, supporting their overall health and development [225, 226].

***Strengths and Limitations***.

This review consolidated global research using 24-h MB guidelines by integrating articles that reported on 24-h MB guidelines in children and adolescents aged 5–17 years. The review’s strengths include its comprehensive, up-to-date data analysis, rigorous methodology. For example, instead of only focusing on one or two behaviours, the included articles reported adherence with all three 24-h MB guidelines together, providing a holistic assessment of daily activity patterns. Several limitations warrant consideration. First, while identifying research gaps, the study was limited by its focus on peer-reviewed journals, which may introduce publication bias [227]. However, this ensures quality through peer reviews. Additionally, only English-language papers were included, possibly overlooking relevant research in other languages, although recent evidence suggests that this has a minimal impact [228]. Second, because the review aimed to provide a broad map of research activity rather than a quantitative synthesis, we did not extract full adjustment sets or effect estimates. Variables labelled as correlates were accepted as defined by the original authors; some of these associations may derive from minimally adjusted analyses and should be interpreted with caution. Future focus-specific systematic reviews should broaden the search to include non-English and grey-literature sources and appraise analytical depth and effect sizes in detail to build on the descriptive foundation provided here.

## Conclusions

Global research on 24-h MB guidelines has expanded since 2016 but remains methodologically modest and concentrated in a handful of higher-income countries. Approximately two thirds of the included articles were conducted on six high- and upper-middle-income countries, whereas only 7% were on low- and middle-income nations. Cross-sectional prevalence investigations predominate; fewer than one in eight articles employed longitudinal designs, about 2% used experimental methods, none achieving a high-quality rating. This concentration restricts external validity and limits causal inference. Future work should extend to under-represented regions, apply rigorous longitudinal and experimental designs with culturally relevant interventions, harmonise measurement protocols, and examine a wider range of outcomes, including cognitive, psychosocial, and academic domains, to build a more robust foundation for policy and practice aimed at improving youth health and well-being worldwide.

## Supplementary Information

Below is the link to the electronic supplementary material.

Supplementary Material 1

Supplementary Material 2

Supplementary Material 3

Supplementary Material 4

Supplementary Material 5

## Data Availability

The data supporting the conclusions of this study have been made available by the corresponding author.

## Abbreviations

24-h MB: 24-hour movement behaviours
ADHD: Attention-deficit/hyperactivity disorder
BEF: Behavioural epidemiology framework
BMI: Body mass index
HDI: Human development index
HRQOL: Health-related quality of life
MB: Movement behaviours
MVPA: Moderate-to-vigorous physical activity
PA: Physical activity
PRISMA: Preferred reporting items for systematic reviews and meta-analyses
PROSPERO: International prospective register of systematic reviews
SB: Sedentary behaviours
ST: Screen time
UK: United Kingdom
USA: United States of America
