# Supplementary material for: Global research on 24-hour movement behaviours guidelines in children and adolescents: a systematic review
Source: Int J Behav Nutr Phys Act. 2025 Aug 8;22:108. doi: 10.1186/s12966-025-01809-5 (PMC12333077; doi:10.1186/s12966-025-01809-5)
Supplement: Supplementary file 3 — Supplementary Material 3 [file 12966_2025_1809_MOESM3_ESM.docx]

**Table 1.** Search strategy in different databases (Primary).

| **Data Base** | **Search strategy** | **Filters** |
| --- | --- | --- |
| Pubmed  (*n* = 6,237) | ((((((((exercise[Title/Abstract]) OR (exercise[Mesh])) OR ("physical activit*"[Title/Abstract]))) OR ("sedentary behavior" [Mesh] OR "screen time" [Mesh] OR sedentar*[Title/Abstract] OR inactiv*[Title/Abstract] OR "screen time"[Title/Abstract] OR "screen based"[Title/Abstract] )) OR ((sleep [Mesh]) OR (sleep*[Title/Abstract]))) OR ("movement*"[Title/Abstract] OR 24-h*[Title/Abstract])) AND (guideline*[Title/Abstract] OR recommendation*[Title/Abstract])) AND (child*[Title/Abstract] OR adolescen*[Title/Abstract] OR young*[Title/Abstract] OR youth*[Title/Abstract] OR student*[Title/Abstract] OR Adolescents [Mesh] OR Child [Mesh]) | Ages Child: 2-18 years;  Since: 2016;  Language: English; |
| Scopus  (*n* = 10,367) | ( ( ( ( ( ( ( ( TITLE-ABS ( exercise ) ) OR ( INDEXTERMS ( exercise ) ) ) OR ( TITLE-ABS ( "physical activit*" ) ) ) ) OR ( INDEXTERMS ( "sedentary behavior" ) OR INDEXTERMS ( "screen time" ) OR TITLE-ABS ( sedentar* ) OR TITLE-ABS ( inactiv* ) OR TITLE-ABS ("screen time") OR TITLE-ABS ( "screen based" ) ) ) OR ( ( INDEXTERMS ( sleep ) ) OR ( TITLE-ABS ( sleep* ) ) ) ) OR ( TITLE-ABS ( movement* ) OR TITLE-ABS ( 24-h* ) ) ) AND ( TITLE-ABS ( guideline* ) OR TITLE-ABS ( recommendation* ) ) ) AND ( TITLE-ABS ( child* ) OR TITLE-ABS ( adolescen* ) OR TITLE-ABS ( young* ) OR TITLE-ABS ( youth* ) OR TITLE-ABS ( student* ) OR INDEXTERMS ( adolescents ) OR INDEXTERMS ( child ) ) | Since: 2016;  Language: English;  Document type: article |
| Web of Science  (*n* = 11,346) | ((TI= (exercise OR "physical activit*" OR inactiv* OR sedentar* OR "screen time" OR "screen based" OR sleep* OR movement* OR 24-h*) AND (TI= (guideline* OR recommendation*))) AND (TI= (child* OR adolescen* OR young* OR youth* OR student*)) OR (AB= (exercise OR "physical activit*" OR sedentar* OR screen* OR inactiv* OR sleep* OR movement* OR 24-h*) AND (AB= (guideline* OR recommendation*))) AND (AB= (child* OR adolescen* OR young* OR youth* OR student*))) | Since: 2016;  Language: English;  Document types: article |
| SPORTDiscus  (n = 1,497) | (((((((((TI "exercise" OR AB "exercise")) OR (DE "exercise")) OR ((TI "physical activit*" OR AB "physical activit*")))) OR (DE "sedentary behavior" OR DE "screen time" OR (TI "sedentar*" OR AB "sedentar*") OR (TI "inactiv*" OR AB "inactiv*") OR (TI "screen time" OR AB "screen time") OR (TI "screen based" OR AB "screen based"))) OR ((DE "sleep") OR ((TI "sleep*" OR AB "sleep*")))) OR ((TI "movement*" OR AB "movement*") OR (TI "24-h*" OR AB "24-h*"))) AND ((TI "guideline*" OR AB "guideline*") OR (TI "recommendation*" OR AB "recommendation*"))) AND ((TI "child*" OR AB "child*") OR (TI "adolescen*" OR AB "adolescen*") OR (TI "young*" OR AB "young*") OR (TI "youth*" OR AB "youth*") OR (TI "student*" OR AB "student*") OR DE "Adolescents" OR DE "Child") | Since: 2016;  Language: English;  Document types: Academic Journals; Age: 2-17 Years |
| APA PsycInfo  (n = 150) | (TI exercise OR AB exercise OR TI "physical activit*" OR AB "physical activit*" OR TI sedentar* OR AB sedentar* OR TI inactiv* OR AB inactiv* OR TI "screen time" OR AB "screen time" OR TI "screen based" OR AB "screen based" OR TI sleep* OR AB sleep* OR TI movement* OR AB movement* OR TI 24-h* OR AB 24-h*) AND (TI guideline* OR AB guideline* OR TI recommendation* OR AB recommendation*) AND (TI child* OR AB child* OR TI adolescen* OR AB adolescen* OR TI young* OR AB young* OR TI youth* OR AB youth* OR TI student* OR AB student*) | Since: 2016;  Language: English;  Document types: Academic Journals;  Ages: 2-17 Years |
| Embase  (n = 3,235) | ((((((((exercise:ti,ab) OR (exercise/exp)) OR ('physical activit*':ti,ab))) OR ('sedentary behavior'/exp OR 'screen time'/exp OR sedentar*:ti,ab OR inactiv*:ti,ab OR 'screen time':ti,ab OR 'screen based':ti,ab)) OR ((sleep/exp) OR (sleep*:ti,ab))) OR (movement*:ti,ab OR 24-h*:ti,ab)) AND (guideline*:ti,ab OR recommendation*:ti,ab)) AND (child*:ti,ab OR adolescen*:ti,ab OR young*:ti,ab OR youth*:ti,ab OR student*:ti,ab OR Adolescents/exp OR Child/exp) | Since: 2016;  Language: English;  Document types: Article;  Ages: 1-17 Years |
