# Supplementary material for: Global research on 24-hour movement behaviours guidelines in children and adolescents: a systematic review
Source: Int J Behav Nutr Phys Act. 2025 Aug 8;22:108. doi: 10.1186/s12966-025-01809-5 (PMC12333077; doi:10.1186/s12966-025-01809-5)
Supplement: Supplementary file 5 — Supplementary Material 5 [file 12966_2025_1809_MOESM5_ESM.docx]

# **Table 1.** Number of articles on 24-hour movement behaviours by country.

| **Country** | **World Bank Classification** | **Number of Articles** | **Percentage Contribution** |
| --- | --- | --- | --- |
| China | Upper-middle-income | 33 | 22.3 |
| Canada | High-income | 30 | 20.3 |
| United States of America (USA) | High-income | 30 | 20.3 |
| Spain | High-income | 16 | 10.8 |
| Australia | High-income | 12 | 8.1 |
| Brazil | Upper-middle-income | 11 | 7.4 |
| Japan | High-income | 6 | 4.1 |
| United Kingdom | High-income | 4 | 2.7 |
| Finland | High-income | 4 | 2.7 |
| Kenya | Lower-middle-income | 4 | 2.7 |
| Portugal | High-income | 4 | 2.7 |
| India | Lower-middle-income | 4 | 2.7 |
| Republic of Korea | High-income | 4 | 2.7 |
| Colombia | Upper-middle-income | 3 | 2.0 |
| South Africa | Upper-middle-income | 3 | 2.0 |
| Hong Kong | High-income | 3 | 2.0 |
| Czechia | High-income | 2 | 1.4 |
| Singapore | High-income | 2 | 1.4 |
| Saudi Arabia | High-income | 2 | 1.4 |
| Switzerland | High-income | 2 | 1.4 |
| Germany | High-income | 2 | 1.4 |
| Thailand | Upper-middle-income | 2 | 1.4 |
| Bangladesh | Lower-middle-income | 2 | 1.4 |
| New Zealand | High-income | 1 | 0.7 |
| Chile | High-income | 1 | 0.7 |
| Taiwan | High-income | 1 | 0.7 |
| Malaysia | Upper-middle-income | 1 | 0.7 |
| Egypt | Lower-middle-income | 1 | 0.7 |
| Mozambique | Low-income | 1 | 0.7 |
| Sri Lanka | Lower-middle-income | 1 | 0.7 |
| Israel | High-income | 1 | 0.7 |
| Mexico | Upper-middle-income | 1 | 0.7 |

***Note:*** If an article included data from multiple countries, that article was counted as research productivity for each of those countries involved, and hence the total is greater than n=148.

**Table 2: Characteristics of the included articles based on their research focus.**

| **Article Characteristics** | **Prevalence and Trends n(%)** | **Correlates n(%)** | **Health and Well-being n(%)** | **Academic Performance n(%)** | **Intervention n(%)** |
| --- | --- | --- | --- | --- | --- |
| **Study Design** | | |  |  |  |
| Cross-sectional | 122 (86.5) | 36 (90.0) | 66 (83.5) | 7 (87.5) | 0 (0) |
| Longitudinal | 15 (10.7) | 3 (7.5) | 10 (12.7) | 1 (12.5) | 0 (0) |
| Longitudinal & Cross-sectional | 3 (2.1) | 1 (2.5) | 3 (3.8) | 0 (0) | 0 (0) |
| Quasi-experimental | 1 (0.7) | 0 (0) | 0 (0) | 0 (0) | 2 (66.7) |
| Experimental | 0 (0) | 0 (0) | 0 (0) | 0 (0) | 1 (33.3) |
| **Physical Activity Measures** | | |  |  |  |
| Self-reported | 111 (78.7) | 31 (77.5) | 32 (40.5) | 7 (87.5) | 2 (66.7) |
| Device-measured | 25 (17.7) | 8 (20.0) | 47 (59.5) | 0 (0) | 0 (0) |
| Both approaches | 5 (3.6) | 1 (2.5) | 0 (0) | 1 (12.5) | 1 (33.3) |
| **Screen Time Measures** | | |  |  |  |
| Self-reported | 137 (97.2) | 39 (97.5) | 79 (100) | 8 (100) | 2 (66.7) |
| Device-measured | 1 (0.7) | 1 (2.5) | 0 (0) | 0 (0) | 0 (0) |
| Both approaches | 3 (2.1) | 0 (0) | 0 (0) | 0 (0) | 1 (33.3) |
| **Sleep Duration Measures** | | |  |  |  |
| Self-reported | 122 (86.5) | 32 (80.0) | 72 (91.1) | 7 (87.5) | 3 (100) |
| Device-measured | 14 (9.9) | 7 (17.5) | 7 (8.9) | 0 (0) | 0 (0) |
| Both approaches | 5 (3.6) | 1 (2.5) | 0 (0) | 1 (12.5) | 0 (0) |
| **Data Type** | | |  |  |  |
| Nationally representative | 48 (34.0) | 23 (57.5) | 22 (27.8) | 1 (12.5) | 0 (0) |
| Non-representative | 93 (66.0) | 17 (42.5) | 57 (72.2) | 7 (87.5) | 3 (100) |

# **Table 3.** Summary of findings of articles focused on prevalence of meeting 24-hour movement behaviours guidelines or time trends (listed in alphabetical order by title of article).

| Title | Summary of Findings | Manuscript Reference Number |
| --- | --- | --- |
| 24 h Activity Guidelines in Children and Adolescents: A Prevalence Survey in Asia-Pacific Cities | The prevalence of meeting all three 24-hour activity guidelines was low across cities in the Asia-Pacific region, ranging from 1.8% to 10.3%. Children from Thiruvananthapuram, India had the highest prevalence at 10.3%, while those from Tokyo, Japan had the lowest at 1.8%. Overall, more boys met all three guidelines compared to girls, with a prevalence of 5.9% for boys and 4.7% for girls. Despite this, adherence to all three activity guidelines was low across all five participating cities. | 169 |
| 24-H movement behaviors and visual impairment among Chinese adolescents with and without obesity | Overall, 28.75% of participants did not comply with any of the 24‐hour movement behaviours guidelines, while only 0.71% followed all three. | 157 |
| 24‑h movement behaviours in Spanish youth before and after 1‑year into the covid‑19 pandemic and its relationship to academic performance | One year into the COVID-19 pandemic, only 0.2% of participants met all three 24‐Hour Movement Guidelines, compared to 3.3% before the pandemic. In contrast, the proportion not meeting any of the guidelines increased significantly from 28.9% pre-pandemic to 66.3% one year into the pandemic. | 110 |
| 24-h Movement Guidelines and Substance Use among Adolescents: A School-Based Cross-Sectional Study | Overall, only 5% of participants met all three recommendations. Boys had a significantly higher prevalence of compliance than girls (7.4% vs. 2.7%), and younger adolescents were more compliant than older ones (7.0% vs. 4.0%). | 103 |
| 24-Hour Movement Behaviors and Impulsivity | Approximately 30% of the sample did not meet any recommendations, whereas only 4.8% met all three movement behaviour recommendations. | 37 |
| 24-Hour Movement Behaviors and Internalizing and Externalizing Behaviors Among Youth | Overall, only 3.9% of participants met all three movement behavior recommendations, while 32.2% met none. | 95 |
| 24-Hour movement behaviours and COVID-19 among children in the Kingdom of Saudi Arabia: A repeat cross-sectional study | At follow-up, only 1.8% of children met all components of the 24‐hour movement guidelines, compared to 3.4% at Time 1. | 122 |
| 24-hour movement behaviours and self-rated health in Chinese adolescents: a questionnaire-based survey in Eastern China | Only 0.9% of adolescents met all three recommendations, while 36% met none. | 142 |
| 24-hour movement guidelines and suicidality among adolescents. | Overall, 5.2% of students met all three recommendations, while 27.7% met none. The prevalence differed significantly by age and gender: 11- to 14-year-old boys had the highest adherence (9.6%), followed by 15- to 20-year-old boys (5.8%), with 15- to 20-year-old girls having the lowest (2.4%). | 94 |
| 24-hour movement guidelines and weight status among preschool-aged children in Bangladesh: A community-level cross-sectional study | Overall, 15% of children met all three 24‐hour movement behavior guidelines, with only minor, non-significant differences observed between boys and girls. | 147 |
| Adherence to 24-h movement behaviour guidelines in families with multiple children | Overall, 8% of families had children meeting all 24‐hour movement guidelines, while 7% had children meeting none. | 139 |
| Adherence to 24-h movement guidelines and cognitive difficulties in adolescents | Only 2.9% of adolescents met all the 24‐hour movement guidelines, while 43.7% met none. | 39 |
| Adherence to 24-h movement guidelines in Spanish schoolchildren and its association with insulin resistance: a cross-sectional study | Overall, 17.6% of participants met all three 24‐hour movement guidelines (15.2% of girls and 20.2% of boys), while 9.1% met none. | 149 |
| Adherence to 24-Hour Movement Guidelines among Spanish Adolescents: Differences between Boys and Girls | Only 5.4% of these adolescents met all three 24-Hour Movement Guidelines, while 10.2% did not meet any of the guidelines. No significant gender differences were observed in the compliance of all three guidelines. | 101 |
| Adherence to 24-hour movement guidelines and academic performance in adolescents | Overall, 5.1% of students met all three movement guidelines, while 39.0% did not meet any. | 23 |
| Adherence to 24-hour movement guidelines and their association with depressive symptoms in adolescents: Evidence from Bangladesh | The percentage of adolescents meeting all three recommendations was 2.2%, while about half did not meet any. | 161 |
| Adherence to 24-Hour Movement Guidelines in Relation to the Risk of Overweight and Obesity Among Children and Adolescents | The percentage of participants meeting exactly all three 24-hour movement guidelines was 6.2%. The prevalence of adherence to all recommendations declined from 9.6% in 2004 to 3.4% in 2015, while the proportion of participants not meeting any guidelines increased from 2.9% to 9.8% over the same period. | 160 |
| Adherence to 24-Hour Movement Recommendations and Health Indicators in Early Adolescence: Cross-Sectional and Longitudinal Associations in the Adolescent Brain Cognitive Development Study | At T1, only 4% of participants met all three 24‐hour movement guidelines, while 31% met none. At T2, 3% met all three guidelines and 43% met none. | 35 |
| Adherence to Combined Healthy Movement Behavior Guidelines among Adolescents: Effects on Cardiometabolic Health Markers | Only 4.8% of adolescents met all three healthy movement behavior guidelines, while 9.3% met none. There were no significant differences between boys and girls in overall compliance. | 17 |
| Adherence to the 24-Hour Movement Guidelines among 10- to 17-year-old Canadians | Only 3% of the sample met all three movement behaviour guidelines, whereas 21% met none. | 56 |
| Adherence to the 24-hour movement guidelines and adiposity in a cohort of at risk youth: A longitudinal analysis | In childhood (8–10 years), 14.2% of participants met all three 24‐hour movement guideline components, while only 0.8% met none; in early adolescence (10–12 years) and adolescence (15–17 years), these figures were 6.1% versus 5.4%, and 0% versus 12.5%, respectively. | 33 |
| Are one-year changes in adherence to the 24-hour movement guidelines associated with depressive symptoms among youth? | Only 2.1% of females and 0.8% of males met the guidelines in both years. More males transitioned to meeting additional guidelines at follow-up compared to females. | 69 |
| Are one-year changes in adherence to the 24-hour movement guidelines associated with flourishing among Canadian youth? | At Year 5, significantly more females (1.4%) met all three guidelines compared to males (1.0%), and at Year 6, the difference remained significant with 0.7% of females versus 0.5% of males meeting all three. | 72 |
| Association between 24‑hour movement behaviors and health‑related quality of life in children | Among children aged 5–13 years, 22.1% met all three guidelines while 8.5% met none; for adolescents aged 14–15 years, 10.7% met all three and 19.1% met none. | 76 |
| Association between 24-hour movement guidelines and physical fitness in children | 9.1% of participants met all three 24‐hour movement guidelines, while 13.2% did not meet any. | 90 |
| Association between air pollution and 24-h movement behaviours in a representative sample of Spanish youth | Overall, 10.7% of young people met the 24‐hour movement guidelines. The prevalence of meeting all three recommendations was highest in areas with low air pollution (14.2%), followed by medium (10.6%) and high (7.6%) air pollution. | 129 |
| Association Between Combinations of 24-Hour Movement Behaviors and Depression Among Adolescents — Inner Mongolia Autonomous Region, China, 2019–2021 | Across three cross-sectional surveys in 2019, 2020, and 2021, only 1.2%, 1.2%, and 2.3% of adolescents, respectively, met all three recommended 24-hour movement guidelines. | 154 |
| Association Between Daily Physical Education Attendance and Meeting 24-Hour Movement Guidelines in Adolescence and Adulthood | Overall, 7.5% of adolescents met all three guidelines. | 150 |
| Association between meeting 24-h movement guidelines and academic performance in a sample of 67,281 Chinese children and adolescents | Overall, 1.7% of participants met all three guidelines, while 28.7% met none. | 145 |
| Association between meeting the 24-h movement guidelines and psychosocial health in children: A cross-sectional study | Overall, 10.2% of participants met all three 24‐hour movement recommendations. | 41 |
| Association between meeting the 24-hour movement guideline and anxiety status in Chinese school-aged adolescents | Only 2.1% of participants met all three 24‐hour movement guidelines, while 26.0% met none. | 152 |
| Association between Physical Activity, Sedentary Behaviors, Sleep, Diet, and Adiposity among Children and Adolescents in China | Overall, 12.5% of participants met the physical activity, sleep, and sedentary behavior recommendations. | 106 |
| Association between the 24-hour movement guidelines and executive function among Chinese children | Overall, 10.1% of participants met all three recommendations, with 16.4% of boys and 3.3% of girls doing so. | 38 |
| Association of Adolescent Bullying Victimization with Meeting 24‑hour Movement Behavior Recommendations: A Cross‑Sectional Study Using the Combined 2015–2019 Youth Risk Behavior Survey | Approximately 4.6% of the full sample met the recommendations for all three 24‐hour movement behaviors. | 124 |
| Associations between 24 hour movement behaviours and global cognition in US children: a cross-sectional observational study | Only 5% of participants met all three recommendations, while 29% met none. | 36 |
| Associations between 24-h Movement Behavior and Internet Addiction in Adolescents: A Cross-Sectional Study | Overall, 25.3% of children did not meet any of the recommended activity behavior guidelines, while only 3.2% met all three. | 127 |
| Associations between 24-h movement behaviors and self-rated health: a representative sample of school-aged children and adolescents in Okinawa, Japan | Elementary school students showed a higher rate of non-adherence (39.2%) compared to junior high school students (10.4%), with only 2.4% of elementary students versus 4.7% of junior high students meeting all three recommendations. | 119 |
| Associations between combinations of 24‐h movement behaviors and physical fitness among Chinese adolescents: Sex and age disparities. | Among 13- to 17-year-olds, 14.0% did not meet any guidelines, while only 3.7% met all three. | 162 |
| Associations between meeting 24-hour movement guidelines and academic achievement in Australian primary school-aged children | According to self-report, 20.3% of children met all three guidelines and 6.1% met none, while accelerometry indicated 12% met all and 11% met none. | 21 |
| Associations between meeting 24-hour movement guidelines and myopia among school-aged children: A cross-sectional study | Only 4.92% of children met all three 24-hour movement behavior guidelines. | 153 |
| Associations between meeting combinations of 24-hour movement recommendations and dietary patterns of children: A 12-country study | Only 7% of children met all three movement behaviour guidelines, and 19% of children did not meet any of the movement behaviour guidelines. | 59 |
| Associations between meeting combinations of 24-h movement guidelines and health-related quality of life in children from 12 countries | Only 7.2% of participants met all three recommendations and 18.9% of the participants met none of the recommendations. | 54 |
| Associations between meeting sleep, physical activity or screen time behaviour guidelines and academic performance in Australian school children | Only 2% of children met all three behaviour guidelines. | 22 |
| Associations between the Canadian 24 h movement guidelines and different types of bullying involvement among adolescents | Overall, 4.9% of students met all three recommendations (7.2% of boys and 2.5% of girls), while 39.4% did not meet any. | 92 |
| Associations of 24-Hour Movement Behavior with Depressive Symptoms and Anxiety in Children: Cross-Sectional Findings from a Chinese Sample | Only 3.2% of participants (boys: 4% vs. girls: 2.2%) met all three 24‐Hour Movement Behavior Guidelines, while 21.9% met none. | 104 |
| Better health indicators of FitSpirit participants meeting 24-h movement guidelines for Canadian children and youth | Only 2.2% of girls met all the recommendations, while 72.5% met one or two, and 25.4% met none. | 84 |
| Canadian 24-h Movement Guidelines, Life Stress, and Self-Esteem Among Adolescents | Overall, 3.5% of participants met all three recommendations, while 44.5% met none. | 128 |
| Canadian children’s and youth’s adherence to the 24-h movement guidelines during the COVID-19 pandemic: A decision tree analysis | During the COVID-19 pandemic, only 2.6% of children and youth met all three movement behavior recommendations. A decision tree analysis revealed that parental confidence in restricting screen time was the strongest predictor: children whose parents strongly believed they could limit screen time and who maintained or increased their walking/biking time were more likely to meet the guidelines (with 16.2% adherence), while those whose parents felt less capable, and older children aged 12–17 in particular, were much less likely to meet them. | 80 |
| Changes in 24-hour movement behaviours during the transition from primary to secondary school among Australian children | The proportion of children meeting the 24-hour integrated movement guidelines declined significantly from 20.5% in their final year of primary school (T1) to 3.6% in their first year of secondary school (T2). The change in adherence to the 24-hour movement behaviors was more significant on weekdays than on weekends. | 171 |
| Changes in Canadian Adolescent Time Use and Movement Guidelines During the Early COVID-19 Outbreak: A Longitudinal Prospective Natural Experiment Design | At Year 8 of COMPASS study, only 2.0% of students in the pre-outbreak group met the combined guidelines for MVPA, sleep, and screen time, compared to just 1.1% (weighted 1.4%) in the early outbreak group. | 113 |
| Changes in Healthy Behaviors and Meeting 24-h Movement Guidelines in Spanish and Brazilian Preschoolers, Children and Adolescents during the COVID-19 Lockdown | Before the lockdown, overall compliance with the 24-hour movement guidelines was very low, 3.0% in Spain and 11.7% in Brazil, which further declined during the lockdown to 0.3% in Spain and 7.5% in Brazil. Among children, Brazilian compliance dropped significantly from 15.3% to 10.2% and spain from 6.3% to NA; while for adolescents the proportions remained unchanged in both Spain (0.3% to 0.3%) and Brazil (1.1% to 1.1%). | 99 |
| Combinations of physical activity, screen time and sleep, and their association with subjective wellbeing in children | Only 1.5% of study participants met all three guidelines and 30% met none. | 137 |
| Compliance of the 24-Hour Movement Guidelines in 9- to 11-Year-Old Children From a Low-Income Town in Chile | Using self-reported data, 3.2% of participants met the 24‐hour movement guidelines, while 27.8% met none. In contrast, when combining accelerometer and questionnaire data, only 0.7% met the guidelines and 36.2% met none. | 96 |
| Compliance With 24-Hour Movement Guidelines in Hong Kong Adolescents: Associations With Weight Status | Only 1.0% of adolescents met all the recommendations, while 38.7% met none. | 74 |
| Compliance with the 24-h movement behaviors guidelines among urban and rural Brazilian preschoolers | Among 5-year-old children, none of the urban children met all three recommendations, while only 9.1% of children in rural areas did. | 164 |
| Compliance with the 24‑h Movement Guidelines for Portuguese children: diferences between boys and girls | Only 3.7% of children met all three 24‐hour Movement Guidelines, while 11.9% met none. There were no significant sex differences, as 4.1% of boys and 3.3% of girls met all three guidelines. | 140 |
| Compliance with the 24-hour movement behavior guidelines and the impact of sleep methods among toddler, preschool, and school-aged children enrolled in the Guelph Family Health Study | 12.9% of school-going children met all three movement behavior guidelines. | 167 |
| Cross-sectional and longitudinal associations of adherence to the 24-hour movement guidelines with mental health problems among Chinese adolescents | Overall, 15.5% of participants met all three guideline recommendations, while 9.2% met none. | 141 |
| Differential Associations Between Meeting 24-Hour Movement Guidelines With Mental Wellbeing and Mental Illness Among Chinese Adolescents | Only 1.7% of participants met recommendations for all three behaviors, while 28.7% met none. | 138 |
| Does compliance with healthy lifestyle behaviours cluster within individuals in Australian primary school‐aged children? | In the Obesity Prevention and Lifestyle data, only 4.3% of participants met all three guidelines, whereas 14.7% met none. In the International Study of Children, Obesity, Lifestyle and Environment, 16.4% met all guidelines and 7.3% met none, while in the National Children's Nutrition and Physical Activity Survey, 15.5% met all guidelines and only 2.6% met none. | 58 |
| Effects of a school-based intervention on physical activity, sleep duration, screen time, and diet in children | Overall, 10% of students met all three recommendations and 14% met none. Among boys, only 1.6% met all three recommendations while 25% met none, whereas among girls, 15.8% met all three and only 1.8% met none. | 126 |
| Environmental tobacco smoke exposure and 24-h movement guidelines in Spanish young people | Overall, 7.3% of the young population met all three 24‐hour movement guidelines. | 155 |
| Factors Associated with Students Meeting Components of Canada’s New 24-Hour Movement Guidelines over Time in the COMPASS Study | Only 1.28% of the sample met the overall 24‐hour Movement Guidelines. | 71 |
| Family history of non-communicable diseases and associations with weight and movement behaviours in Australian school-aged children: a prospective study | Only 11% of children met the combined movement behaviour guidelines, with a significant difference between boys (13.3%) and girls (9%). | 60 |
| Few Canadian children and youth were meeting the 24-hour movement behaviour guidelines 6-months into the COVID-19 pandemic: Follow-up from a national study | During the second wave, 4.5% of children(5-11 Years) (4.6% girls, 4.3% boys) and 1.9% of youth (12-17 Years) (1.3% girls, 2.4% boys) met the movement guidelines, yielding an overall compliance rate of 3.1%. In the first wave, 4.8% of children (2.8% girls, 6.5% boys) and 0.6% of youth (0.8% girls, 0.5% boys) met the combined guidelines, or 2.6% overall. | 107 |
| Health associations with meeting new 24-h movement guidelines for Canadian children and youth | Overall, 17.1% of children met all three guidelines, while 11% met none. | 31 |
| Healthy movement behaviours in children and youth during the COVID-19 pandemic: Exploring the role of the neighbourhood environment | Children and youth with greater outdoor opportunities, such as those living in houses with access to parks, tended to cluster into the increased outdoor activities group, where 4.9% met the 24‐hour movement guidelines. In contrast, barriers like high dwelling density and proximity to major streets led to membership in the decreased outdoor activities group, where only 1.0% met the guidelines, highlighting the relationship of the built environment on movement behaviours compliance. | 78 |
| Healthy or Unhealthy? The Cocktail of Health-Related Behavior Profiles in Spanish Adolescents | Only 1.7% of the sample met all three guidelines, whereas 8.9% did not meet any of the guidelines. | 67 |
| Identifying Risk Profiles for Nonadherence to the 24-Hour Movement Guidelines for Children and Youth 6 Months Into the COVID-19 Pandemic | Only 3.8% of children and youth met all three movement behavior recommendations. For example, among children whose parents reported high capability to restrict screen time, only 1.4% met the recommendations if their outdoor time decreased during the pandemic, compared to 9.6% if it remained the same and 22.2% if it increased. In contrast, among children whose parents reported low capability, only 1.7% met the recommendations if their families did not use online resources, versus 5.8% if they did. | 135 |
| Impact of the COVID-19 virus outbreak on 24-h movement behaviours among children in Saudi Arabia: A cross-sectional survey | Only 3.4% of Saudi children met all components of the 24‐hour movement guidelines, with a slightly higher prevalence among girls (3.8%) than boys (2.7%). | 121 |
| Impact of the COVID-19 virus outbreak on movement and play behaviours of Canadian children and youth: a national survey | Only 4.8% of children (2.8% of girls, 6.5% of boys) and 0.6% of youth (0.8% of girls, 0.5% of boys) met the combined movement behaviour guidelines during COVID-19 restrictions. | 79 |
| Impact of Virtual vs. In-Person School on Children Meeting the 24-h Movement Guidelines during the COVID-19 Pandemic | elementary-age children met all the movement behaviours guidelines on a significantly higher proportion of days during in-person schooling than during virtual schooling. For example, in Kindergarten, children met the guidelines on 26.0% of days in-person versus 10.2% during virtual classes, with similar trends observed across grades. | 118 |
| Is adherence to 24-Hour Movement Guidelines associated with a higher academic achievement among adolescent males and females? | Prevalence of meeting all three guidelines was 5.8% (5.0% girls and 6.5% boys) | 100 |
| Is adherence to the 24-hour movement guidelines associated with a reduced risk of adiposity among children and adolescents? | Among children (8–13 years), 6.5% met all three recommendations while 33% met none, whereas among adolescents (14–18 years), only 2.2% met all three and 36.4% met none. | 73 |
| Is adherence to the 24-Hour Movement Guidelines associated with Mediterranean dietary patterns in adolescents? | Overall, 9.9% of participants did not meet any recommendation, while 5.6% met all three 24‐Hour Movement Guidelines. | 109 |
| Is adherence to the Canadian 24-Hour Movement Behaviour Guidelines for Children and Youth associated with improved indicators of physical, mental, and social health? | Only 2.6% of participants met all three movement behaviour guidelines, while 21.4% met none. | 32 |
| Is meeting with the 24-h movement recommendations linked with suicidality? Results from a nationwide sample of 44,734 U.S. adolescents | Overall, only 2.1% of participants met all three 24-hour movement recommendations. | 158 |
| Levels and correlates of 24-hour movement behaviors among South Koreans: Results from the Korea National Health and Nutrition Examination Surveys, 2014 and 2015 | Among South Korean youth aged 12–17, only 3.2% achieved the most ideal combination of 24‐hour movement behaviours, while 14.1% met none of the guidelines. | 61 |
| Lifestyle Behaviours of Children and Adolescents During the First Two Waves of the COVID-19 Pandemic in Switzerland and Their Relation to Well-Being: An Observational Study | For children aged 5–9 years, 53.6% met all three recommendations before the pandemic, which dropped to 33.7% during lockdown and increased to 41.1% during the second wave. For 10–12-year-olds, the figures were 53.4% before the pandemic, 24.9% during lockdown, and 34.0% in the second wave. Among adolescents aged 13–16 years, only 19.3% met all three recommendations before the pandemic, falling to 6.2% during lockdown and rising slightly to 8.9% during the second wave. | 116 |
| Longitudinal and cross-sectional associations of adherence to 24-hour movement guidelines with cardiometabolic risk | At baseline, 52.5% of children met all three 24‐hour movement guidelines, but only 24.9% of children did so at 2-year follow-up. | 97 |
| Meeting 24 h Movement Guidelines and Health-Related Quality of Life in Youths during the COVID-19 Lockdown | Only 3.5% of participants met all three 24‐hour movement guidelines. | 111 |
| Meeting 24-h movement guidelines and associations with health related quality of life of Australian adolescents | Only 2.4% of adolescents met all three recommendations, while 18% met none. | 68 |
| Meeting 24-h Movement Guidelines is Related to Better Academic Achievement: Findings from the YRBS 2019 Cycle | Only 2.8% of adolescents met all the 24-hour movement behavior guidelines, while 42.6% met none. | 108 |
| Meeting 24-h movement guidelines: Prevalence, correlates, and associations with socioemotional behavior in Spanish minors | Overall, 13.5% of participants met all three movement guidelines. | 130 |
| Meeting 24-h movement guidelines: Prevalence, correlates, and the relationships with overweight and obesity among Chinese children and adolescents | Only 5.12% of Chinese children and adolescents met the 24‐hour movement guidelines. | 19 |
| Meeting 24-Hour Movement and Dietary Guidelines: Prevalence, Correlates and Association with Weight Status among Children and Adolescents: A National Cross-Sectional Study in China | Only 2.1% of children met the full 24‐hour movement guidelines, with significant differences observed by sex and age. | 131 |
| Meeting 24-Hour Movement Guidelines for Children and Youth and associations with psychological well-being among South Korean adolescents | Only 1.6% of adolescents met the combined guidelines, while 18.1% did not meet any of the recommendations. | 62 |
| Meeting the Canadian 24-Hour Movement Guidelines and physical-mental comorbidity among Chinese children and adolescents: Prevalence, associations, and the population impacts | Only 3.8% of children met all three recommendations, while 17.0% met none. | 156 |
| Meeting the Canadian 24-Hour Movement Guidelines for Children and Youth | Overall, 17.5% of children and adolescents met all three 24‐Hour Movement Behaviours Guidelines. A higher proportion of children aged 5–11 (29.6%) met these guidelines compared to youth aged 12–17 (5.5%), with boys (22.9%) exceeding girls (11.8%). | 55 |
| Moderate–Vigorous Physical Activity, Screen Time and Sleep Time Profiles: A Cluster Analysis in Spanish Adolescents | Only 28.21% of young people met the recommendations for 24-hour movement behaviours guidelines. | 134 |
| Movement and mental health: Behavioral correlates of anxiety and depression among children of 6–17 years old in the U.S. | Overall, 20.9% of children aged 6–11 years and 24.9% of adolescents aged 12–17 years met none of the three 24‐hour movement guidelines, whereas only 7.2% and 2.0%, respectively, met all three. | 66 |
| Movement and Play Behaviors of Egyptian Children After Covid-19 Pandemic Restrictions: Cross-Sectional Study | Overall, only 2.3% of screened children fulfilled the 24‐hour combined movement behavior criteria, with 2.5% of 5- to 9-year-olds meeting the criteria compared to 1.7% of 10- to 14-year-olds. | 123 |
| Movement Behaviors and Perceived Loneliness and Sadness within Alaskan Adolescents | Approximately 5% of the sample met recommendations for all three movement behaviors. | 89 |
| Movement in High School: Proportion of Chinese Adolescents Meeting 24-Hour Movement Guidelines | Overall, only 0.3% of the sample met all three guidelines, while 5.1% met none. | 77 |
| One size does not fit all: identifying clusters of physical activity, screen time, and sleep behaviour co-development from childhood to adolescence | Between 10% and 39% of youth did not meet any recommendations at various data collection cycles. | 88 |
| Parents’ Perceptions of Changes in Sleep Duration, Physical Activity, and Sedentary Behavior in Arab Israeli Children during the COVID-19 Outbreak | Meeting the 24-hour movement guidelines significantly deteriorated during the COVID-19 outbreak compared to before the pandemic among children aged 5-13 years. The prevalence decreased from 16.4% to 3.7%. | 170 |
| Physical activity, screen time and sleep duration: Combined associations with psychosocial health among Canadian children and youth | Overall adherence to all three 24-Hour Guidelines was low, with 13.9% of children and just 4.8% of youth meeting them. | 40 |
| Physical activity, screen time, and sleep: do German children and adolescents meet the movement guidelines? | Among respondents, 9.7% met all three guidelines, with the highest prevalence among 9- to 10-year-olds (23%) and only 1.7% among 16- to 18-year-olds. Approximately 25% did not meet any of the guidelines. | 114 |
| Prevalence and correlates of adherence to movement guidelines among urban and rural children in Mozambique: a cross-sectional study | Only 10.8% of the entire sample met all three 24‐hour movement guidelines, with a segmented prevalence of 17.7% among rural children compared to just 3.6% among urban children. | 65 |
| Prevalence and correlates of adherence to the combined movement guidelines among Czech children and adolescents | Approximately 6.5% of children and 2.2% of adolescents met all of the combined movement guidelines. | 86 |
| Prevalence and correlates of compliance with 24-h movement guidelines among children from urban and rural Kenya—The Kenya-LINX project | Overall, only 7% of children met the combined movement guidelines, with higher compliance in rural areas (10%) compared to urban areas (5%). | 117 |
| Prevalence and correlates of meeting physical activity, screen time, and sleep duration guidelines among Brazilian adolescents: findings from ERICA | Only 8.7% of adolescents met all three movement guidelines (girls: 6.8%; boys: 10.6%), while 19.4% met none. | 115 |
| Prevalence and correlates of meeting sleep, screen-time, and physical activity guidelines among adolescents in the United Kingdom | Only 9.7% of adolescents met the recommendations for sleep, screen time, and moderate-to-vigorous physical activity concurrently. | 63 |
| Prevalence and Likelihood of Meeting Sleep, Physical Activity, and Screen-Time Guidelines Among US Youth | Overall, just 5% of US adolescents met the recommendations for sleep, physical activity, and screen time concurrently, with only 3% of girls meeting the guidelines compared to 7% of boys. | 64 |
| Prevalence and Selected Sociodemographic of Movement Behaviors in Schoolchildren from Low and Middle-Income Families in Nanjing, China: A Cross-Sectional Questionnaire Survey | Only 2.9% of participants met the combined guidelines. | 81 |
| Prevalence and sociodemographic factors associated with meeting the 24-hour movement guidelines in a sample of Brazilian adolescents | According to self-reports, only 3% of children met all three movement behaviour recommendations (with 32.97% meeting none), while accelerometer data showed that just 0.2% met all three (with 45.49% meeting none). | 75 |
| Prevalence and trends in Australian  adolescents’ adherence to 24-hour movement  guidelines: findings from a repeated national  cross-sectional survey | Compliance with all three guidelines remained very low (under 3%) across all survey rounds. | 91 |
| Prevalence of movement behaviors in Mexico. | Only 1.5% of children and 4.7% of adolescents in Mexico met the recommendations for all three movement behaviors. | 168 |
| Proportion and Correlates of Children in the US-Affiliated Pacific Region Meeting Sleep, Screen Time, and Physical Activity Guidelines | Twenty-seven percent of children met all integrated guidelines. | 163 |
| Proportion of children meeting recommendations for 24-hour movement guidelines and associations with adiposity in a 12-country study | Across 12 countries, only 7% of children adhered to all three movement behaviour guidelines, with the highest adherence observed in Australia and Canada. | 30 |
| Proportion of Chinese Children and Adolescents Meeting 24-Hour Movement Guidelines and Associations with Overweight and Obesity | Overall, 7.3% of Chinese children and adolescents met the overall 24‐Hour Movement Guidelines, with boys showing a higher prevalence (11.8%) than girls (3.4%) and a noticeable decline with increasing age. | 20 |
| Proportion of Japanese primary school children meeting recommendations for 24-h movement guidelines and associations with weight status | 10.5% met all three recommendations while 13.2% met none. | 87 |
| Prospective associations between adherence to 24-hour movement guidelines and mental well-being in Chinese adolescents | Only 8.1% of participants met all three recommendations at baseline, while 17% met none. | 148 |
| Recommendations on screen time, sleep and physical activity: associations with academic achievement in Swiss adolescents | Overall, 7.5% of students did not meet any recommendations, while 30.1% met all three. | 98 |
| Regional Socioeconomic Deprivation in Germany and Adherence to the 24-h Movement Guidelines among Children and Adolescents | Only 2.3% of students met all guidelines, while nearly half (48.7%) met none. | 146 |
| Relationship Between Meeting 24-Hour Movement Guidelines and Cardiometabolic Risk Factors in Children | Only 8.4% of the sample met all three guidelines and 26.9% met none of the guidelines. | 57 |
| Relationship of 24-Hour Movement Behaviors with Weight Status and Body Composition in Chinese Primary School Children: A Cross-Sectional Study | Overall, 26.1% of participants met all three behavioral recommendations, while only 5.9% met none. | 12o |
| School-aged children’s movement behaviours and subjective health complaints in Japan: a cross-sectional study during COVID-19 pandemic-related school closures and after school reopenings | During school closure, 3.9% of boys adhered to all three movement behaviors, while 4.8% adhered during school reopening. Among girls, 2.9% adhered during school closure, and 3.3% adhered during school reopening. | 165 |
| Secular trends in the prevalence of meeting 24-hour movement guidelines among U.S. adolescents: evidence from NHANES 2007–2016 | Overall, 24.5% of participants met all three 24-hour movement recommendations, while 6.5% met none. A higher proportion of boys (29.3%) met all three recommendations compared to girls (18.7%). The prevalence of meeting all three recommendations declined from 24.9% in the 2007–2008 cycle to 19.2% in the 2015–2016 cycle, reflecting a 5.7% decrease. Subgroups with the highest prevalence of adherence included non-Hispanic White participants (25.1%), those with a poverty-income ratio below 1.0 (28.3%), individuals with an appropriate total energy intake (27.8%), and those whose parents had more than 12 years of education (42.4%). | 159 |
| Sexual identity-behavior discordance and meeting 24-hour movement behavior recommendations in adolescents | Overall, only 4.4% of participants met all three 24-hour movement behaviours recommendations. | 151 |
| Six-year trends and intersectional correlates of meeting 24-Hour Movement Guidelines among South Korean adolescents: Korea Youth Risk Behavior Surveys, 2013-2018 | Only 0.5% of children met all three recommendations on weekdays and 0.8% on weekends. | 85 |
| Sleep, sedentary behavior, and physical activity in Brazilian adolescents: Achievement recommendations and BMI associations through compositional data analysis | No adolescent in the study was able to achieve all of the daily 24-hour movement recommendations. | 172 |
| Sociodemographic diferences in 24-hour time-use behaviours in New Zealand children | Overall, 10.6% of children met the combined 24‐hour Movement Guidelines. | 133 |
| Survey of the Adequacy of Brazilian Children and Adolescents to the 24-Hour Movement Guidelines before and during the COVID-19 Pandemic | Before the pandemic, 39.5% of children aged 6 to 17 met the guidelines, but during the pandemic this dropped dramatically to 4.94%, a difference that was statistically significant | 136 |
| Thai Preschoolers’ movement behaviors outside kindergarten: prevalence of meeting individual and integrated movement guidelines | 14.6% of children met the integrated movement guidelines, while 10.8% did not meet any of the three guidelines. | 166 |
| The Association of Soft Drink Consumption and the 24-Hour Movement Guidelines with Suicidality among Adolescents of the United States | Overall, only 3.1% of adolescents met all the recommendations (4.3% of boys and 1.9% of girls). | 125 |
| The Associations between Meeting 24-Hour Movement Guidelines (24-HMG) and Mental Health in Adolescents—Cross Sectional Evidence from China | Overall, only 0.71% of adolescents met all three recommendations (1.03% for boys and 0.33% for girls), while 28.70% met none. | 18 |
| The associations between meeting 24-hour movement guidelines and adiposity in Asian Adolescents: The Asia-Fit Study | With the exception of adolescents in Shanghai (3%), less than 1% of adolescents in the other seven cities met all three guidelines, and except in Bangkok, Shanghai, and Tokyo, 50% or more of children did not meet any guidelines in other cities. | 34 |
| The Canadian 24-Hour Movement Guidelines and Psychological Distress among Adolescents | Only 4.7% of participants met all three recommendations, while 38.9% met none. | 93 |
| The Canadian 24‑hour movement guidelines and self‑rated physical and mental health among adolescents | Overall, meeting all three recommendations was reported by 5.0% of the sample in 2017 and by 3.5% in 2019, while 39.9% and 44.5% met none, respectively. | 102 |
| The Combinations of Physical Activity, Screen Time, and Sleep, and Their Associations with Self-Reported Physical Fitness in Children and Adolescents | Only 0.9% of participants met the 24‐hour movement guidelines, while 36% met none. | 112 |
| The neighbourhood social environment correlates with meeting 24-h movement behaviour recommendations in females: a cross-sectional study using the 2019 National Survey of Children’s Health | Only 12.5% of participants met all the 24‐hour movement behavior recommendations. | 105 |
| Trajectories of 24-h movement guidelines from middle adolescence to adulthood on depression and suicidal ideation: a 22-year follow-up study | Overall, 7.7% of adolescents met all three guidelines, while 17.2% met none. | 8 |
| Trends in adherence to the 24-h movement guidelines among US adolescents from 2011 to 2019: Evidence from repeated cross-sectional cycles of the Youth Risk Behavior Surveillance System | Overall, 3.3% of adolescents met the 24‐hour movement guidelines. There was a clear downward trend in adherence from 3.6% in 2011 to 2.6% in 2019, with similar declines seen among females and Black/African American adolescents. | 144 |
| Twenty-Four-Hour Movement Guidelines and Body Weight in Youth | Overall, 9.4% of children met all three 24-hour healthy movement guidelines. | 82 |
| Twenty‑four‑hour movement guidelines during adolescence and its association with obesity at adulthood: results from a nationally representative study | Only 7.4% of adolescents met all three recommendations, while 18.7% met none. | 10 |
| Twenty-four-hour movement guidelines during middle adolescence and their association with glucose outcomes and type 2 diabetes mellitus in adulthood | Only 2.1% of adolescents met all three guidelines, while 37.8% met none. | 9 |
| U.S. Children Meeting Physical Activity, Screen Time, and Sleep Guidelines | Only 8.8% of U.S. children met all three guidelines combined. Moreover, the prevalence declined significantly with age. | 83 |
| Weight status and meeting the physical activity, sleep, and screen-time guidelines among Texas children: results from a population based, cross-sectional analysis | A greater proportion of healthy weight children (9.9% overall; 8.3% of girls and 11.3% of boys) met all three guidelines concurrently compared to thin (3.3%), overweight (5.7%), obese (3.5%), and morbidly obese (1.0%) children. | 132 |
| Weight status, body composition, and diet quality of Spanish schoolchildren according to their level of adherence to the 24-hour movement guidelines | Only 15% of schoolchildren met all three guidelines. | 70 |
| What is the role of adherence to 24-hour movement guidelines in relation to physical fitness components among adolescents? | Only 5.7% of participants met all three recommendations, while 9.9% met none. | 143 |

# **Table 4.** Summary of findings of articles focused on correlates of adherence with 24-hour movement behaviours guidelines (listed in alphabetical order by title of the article).

| Title | Summary of Findings | Manuscript Reference Number |
| --- | --- | --- |
| 24-hour movement guidelines and weight status among preschool-aged children in Bangladesh: A community-level cross-sectional study | Overall, 15% of children met all three 24‐hour movement behavior guidelines, with only minor, non-significant differences observed between boys and girls. Notably, children attending Noorani (Islamic) schools were twice as likely to meet all three recommendations compared to those in secular schools (Table 4). Additionally, adherence to all three guidelines was not associated with overweight or obesity. | 147 |
| Adherence to 24-h movement behaviour guidelines in families with multiple children | Compared to families with children in different age‐based sleep guideline categories, families with children in the same age‐based sleep guideline category had nearly double (Table 3) the odds of having all children meet the guidelines. | 139 |
| Association between air pollution and 24-h movement behaviours in a representative sample of Spanish youth | Compared with those living in low air pollution areas, individuals in medium and high air pollution areas had lower odds of meeting the guidelines (with odds ratios of 0.55 and 0.45, respectively) (Figure 3). | 129 |
| Association Between Daily Physical Education Attendance and Meeting 24-Hour Movement Guidelines in Adolescence and Adulthood | Adolescents who participated in daily physical education lessons (PEL) had a higher likelihood of meeting all three guidelines compared to those who did not attend any PELs, about 1.7 times higher for boys and nearly 3.8 times higher for girls. In addition, each extra hour per week of PEL participation increased the likelihood of meeting all three recommendations by approximately 11% for boys and 67% for girls (Table 2) | 150 |
| Canadian children’s and youth’s adherence to the 24-h movement guidelines during the COVID-19 pandemic: A decision tree analysis | A decision tree analysis revealed that parental confidence in restricting screen time was the strongest predictor: children whose parents strongly believed they could limit screen time and who maintained or increased their walking/biking time were more likely to meet the guidelines (with 16.2% adherence), while those whose parents felt less capable, and older children aged 12–17 in particular, were much less likely to meet them (Figure 1). | 80 |
| Environmental tobacco smoke exposure and 24-h movement guidelines in Spanish young people | Participants exposed to environmental tobacco smoke were 37% less likely to meet all three guidelines (Figure 2). | 155 |
| Factors Associated with Students Meeting Components of Canada’s New 24-Hour Movement Guidelines over Time in the COMPASS Study | Beginning participation in community sports after grade 9 was the only student-level characteristic associated with a higher likelihood of meeting all the guidelines (Table 6). | 71 |
| Family history of non-communicable diseases and associations with weight and movement behaviours in Australian school-aged children: a prospective study | Boys with a family history of type 2 diabetes had 43% lower odds of meeting the combined guidelines compared to those without such a history (Figure 2). | 60 |
| Few Canadian children and youth were meeting the 24-hour movement behaviour guidelines 6-months into the COVID-19 pandemic: Follow-up from a national study | Parental support was positively correlated with their child's movement behaviors at both time points (Abstract). | 107 |
| Healthy movement behaviours in children and youth during the COVID-19 pandemic: Exploring the role of the neighbourhood environment | Children and youth with greater outdoor opportunities, such as those living in houses with access to parks, tended to cluster into the increased outdoor activities group, where 4.9% met the 24‐hour movement guidelines. In contrast, barriers like high dwelling density and proximity to major streets led to membership in the decreased outdoor activities group, where only 1.0% met the guidelines, highlighting the relationship of the built environment on movement behaviours compliance (Table 3). | 78 |
| Identifying Risk Profiles for Nonadherence to the 24-Hour Movement Guidelines for Children and Youth 6 Months Into the COVID-19 Pandemic | The analysis showed that the strongest predictor of meeting all three was the parental perceived capability to restrict screen time, followed by the use of online resources to support movement behaviors and changes in the time spent outdoors. For example, among children whose parents reported high capability to restrict screen time, only 1.4% met the recommendations if their outdoor time decreased during the pandemic, compared to 9.6% if it remained the same and 22.2% if it increased. In contrast, among children whose parents reported low capability, only 1.7% met the recommendations if their families did not use online resources, versus 5.8% if they did (Figure 1). | 135 |
| Impact of the COVID-19 virus outbreak on 24-h movement behaviours among children in Saudi Arabia: A cross-sectional survey | Children of older parents, as well as those with lower parental education levels and monthly incomes, were more likely to meet all the guidelines (Table 4 and 5). | 121 |
| Impact of the COVID-19 virus outbreak on movement and play behaviours of Canadian children and youth: a national survey | Parental encouragement and support, engagement in physical activity, and family dog ownership were positively associated with healthier movement behaviours (Abstract). | 79 |
| Impact of Virtual vs. In-Person School on Children Meeting the 24-h Movement Guidelines during the COVID-19 Pandemic | Overall, the odds of meeting the 24‐hour movement guidelines were 1.70 times higher during in-person schooling compared to virtual schooling (Table 2). | 118 |
| Integrating perceived physical environments and the theory of planned behaviors when explaining adherence to 24-hour movement guidelines in Chinese adolescents | Perceived physical environment and Theory of Planned Behaviour (TPB) constructs were associated with adolescents' adherence to 24-hour movement behaviour guidelines. Habit strength had a direct association with adherence, while past behaviour showed a significant association with adherence (Abstract). | 174 |
| Levels and correlates of 24-hour movement behaviors among South Koreans: Results from the Korea National Health and Nutrition Examination Surveys, 2014 and 2015 | Older adolescents, females, and those residing in metro Seoul generally exhibited less favourable movement behaviour patterns (Abstract). | 61 |
| Longitudinal and cross-sectional associations of adherence to 24-hour movement guidelines with cardiometabolic risk | Those meeting all three at baseline having 3.4 times higher odds of continued adherence (Section 3.1). | 97 |
| Meeting 24-h movement guidelines: Prevalence, correlates, and associations with socioemotional behavior in Spanish minors | Adherence to all three guidelines was more likely among children, those with higher socioeconomic status, higher human development indices, lower income inequality, and better diet quality (Table 2). | 130 |
| Meeting 24-h movement guidelines: Prevalence, correlates, and the relationships with overweight and obesity among Chinese children and adolescents | Older children and adolescents were less likely to meet the guidelines, and higher parental education and family income were linked to better adherence (Abstract). | 19 |
| Meeting 24-Hour Movement and Dietary Guidelines: Prevalence, Correlates and Association with Weight Status among Children and Adolescents: A National Cross-Sectional Study in China | Grade, sex, residence, mother’s age, and father’s education level were all significant correlates (Figure 3). | 131 |
| Physical activity, screen time, and sleep: do German children and adolescents meet the movement guidelines? | Adherence with all three behaviours was associated with younger age, male gender, higher self-reported socioeconomic status, and school type, as well as with better subjective school performance, less frequent ADHD, lower depressive symptoms and BMI, and no recent substance use (Table 2). | 114 |
| Prevalence and correlates of adherence to movement guidelines among urban and rural children in Mozambique: a cross-sectional study | Children with higher parental education were 63% less likely to meet the guidelines; children from urban schools were 79% less likely to meet the guidelines compared to rural school children; and longer outdoor time was associated with 33% lower likelihood of meeting the recommendations (Table 4). | 65 |
| Prevalence and correlates of adherence to the combined movement guidelines among Czech children and adolescents | Among children, girls were significantly less likely to meet the guidelines than boys (about 60% lower odds), and those with overweight or obese fathers had around 70% lower odds of adherence compared to those with normal-weight fathers (Table 5). | 86 |
| Prevalence and correlates of compliance with 24-h movement guidelines among children from urban and rural Kenya—The Kenya-LINX project | The likelihood of meeting the guidelines decreased with age, while children who could swim and those who did not engage in screen time before school were more likely to meet the recommendations (Table 2). | 117 |
| Prevalence and correlates of meeting physical activity, screen time, and sleep duration guidelines among Brazilian adolescents: findings from ERICA | Male sex, being 14–15 years old, having black or brown skin, attending school in the afternoon, and living in the less developed Northern and Northeastern regions were linked with a higher prevalence of meeting all three guidelines (Table 3). | 115 |
| Prevalence and correlates of meeting sleep, screen-time, and physical activity guidelines among adolescents in the United Kingdom | Compared to girls from the lowest income tertile, those in the highest were more than twice as likely to meet all three guidelines, whereas girls with depressive symptoms were less likely to do so compared to their peers without these symptoms. Similarly, compared to boys with a normal body mass index, obese boys were less likely to meet all three recommendations, and boys with depressive symptoms were also less likely compared to those without (Table). | 63 |
| Prevalence and Likelihood of Meeting Sleep, Physical Activity, and Screen-Time Guidelines Among US Youth | Compared to younger adolescents (14 years and under) and non-Hispanic white peers, older teens, non-white adolescents, those who were overweight or obese, and those using marijuana or reporting depressive symptoms were less likely to meet all three recommendations, and girls who used alcohol were 28% less likely to meet them compared to girls who did not (Table). | 64 |
| Prevalence and Selected Sociodemographic of Movement Behaviors in Schoolchildren from Low and Middle-Income Families in Nanjing, China: A Cross-Sectional Questionnaire Survey | Both residential area and grade level were significant correlates of adherence to the overall 24‐hour movement guidelines (Table 3). | 81 |
| Prevalence and sociodemographic factors associated with meeting the 24-hour movement guidelines in a sample of Brazilian adolescents | Sex, age, socioeconomic status, household size, family structure, and parental higher education were not significantly associated with meeting all three behaviours (Table 3). | 75 |
| Prevalence and trends in Australian  adolescents’ adherence to 24-hour movement  guidelines: findings from a repeated national  cross-sectional survey | Some apparent differences were observed by sex, grade level, and socio-economic area, these variations were not statistically significant (Table 2). | 91 |
| Proportion and Correlates of Children in the US-Affiliated Pacific Region Meeting Sleep, Screen Time, and Physical Activity Guidelines | Females were 1.5 times more likely to meet all recommendations than males. Children aged 8 years were 57% less likely to meet all recommendations compared to those aged 5 years. Those living in lower-middle-income jurisdictions were 2.3 times more likely to meet all recommendations compared to those from high-income jurisdictions. Overweight and obese children were 53% and 58% less likely, respectively, to meet all three recommendations compared to their healthy-weight peers. Children whose caregivers completed high school or attended college were about 50% less likely to meet all recommendations compared to those whose caregivers did not complete high school. Children from households earning between $10,000–35,000 per year were 40% less likely to meet all recommendations compared to those from households earning less than $10,000 per year (Table 2). | 163 |
| Regional Socioeconomic Deprivation in Germany and Adherence to the 24-h Movement Guidelines among Children and Adolescents | Students from the most deprived communities were half as likely to meet all three guidelines compared to those from the most affluent regions. students who met all three guidelines were significantly more likely to be male, younger, and to report a higher perceived social status compared to those who did not meet all three guidelines (Abstract). | 146 |
| Sexual identity-behavior discordance and meeting 24-hour movement behavior recommendations in adolescents | Although the prevalence of meeting all three 24-h MB guidelines was reported, and the correlates of meeting individual guidelines were documented, the correlates of meeting all three guidelines together were not reported (Abstract). | 151 |
| Six-year trends and intersectional correlates of meeting 24-Hour Movement Guidelines among South Korean adolescents: Korea Youth Risk Behavior Surveys, 2013-2018 | Male adolescents, regardless of social class, were more likely to meet all three recommendations compared to females in the lowest social class, although females from higher socio economic backgrounds also were more likely to comply with all movement behaviours compared to lower (Table 4). | 85 |
| Sociodemographic diferences in 24-hour time-use behaviours in New Zealand children | Child ethnicity and mother’s education were significantly associated with adherence to these guidelines (Table 4). | 133 |
| Thai Preschoolers’ movement behaviors outside kindergarten: prevalence of meeting individual and integrated movement guidelines | Age was negatively associated with meeting the integrated movement guidelines, meaning that older children were less likely to meet the recommended guidelines (Table 4). | 166 |
| The neighbourhood social environment correlates with meeting 24-h movement behaviour recommendations in females: a cross-sectional study using the 2019 National Survey of Children’s Health | Knowing where to go for help was associated with a 2.05 times higher probability of meeting the recommendations, and having a safe school environment was associated with a 1.90 times higher probability; these associations remained significant among females (Table 2). | 105 |
| Typologies of Family Functioning and 24-h Movement Behaviors | Compared to children from families with high acceptance, high monitoring, and low conflict (P1), those from families with H-acceptance, M-monitoring, M-conflict (P2) had 46% lower odds, those with H-acceptance, M-monitoring, H-conflict (P3) had 72% lower odds, those with L-acceptance, L-monitoring, M-conflict (P4) had 76% lower odds, and those with M-acceptance, L-monitoring, H-conflict (P5) had 81% lower odds of meeting all three movement behavior recommendations (Table 3). | 173 |
| U.S. Children Meeting Physical Activity, Screen Time, and Sleep Guidelines | Being female (versus male); an adolescent (versus child); black, Asian, or other race (versus white race); and underweight, overweight, or obese (versus normal weight) were all significantly associated with a lower likelihood of meeting all the 3 guidelines (Table 2). | 83 |
| Weight status and meeting the physical activity, sleep, and screen-time guidelines among Texas children: results from a population based, cross-sectional analysis | Children who were thin, overweight, obese, or morbidly obese had lower odds of meeting all three guidelines compared to healthy weight children (Table 3). | 132 |

# **Table 5.** Summary of findings of articles focused on health and well-being outcomes associated with compliance with 24-hour movement behaviours guidelines (listed in alphabetical order according to article title).

| Title | Summary of Findings | Manuscript Reference Number |
| --- | --- | --- |
| 24-H movement behaviors and visual impairment among Chinese adolescents with and without obesity | Adherence to these guidelines was not significantly associated with visual impairment in adolescents compared to non-adherence. | 157 |
| 24-h Movement Guidelines and Substance Use among Adolescents: A School-Based Cross-Sectional Study | Additionally, among girls, meeting all three recommendations was associated with lower odds of cannabis use compared to those meeting none. | 103 |
| 24-Hour Movement Behaviors and Impulsivity | Compared to meeting none, meeting all three was associated with lower positive urgency, lower negative urgency, lower behavioural inhibition, greater perseverance, and better delay-discounting scores. | 37 |
| 24-Hour Movement Behaviors and Internalizing and Externalizing Behaviors Among Youth | Meeting all three was associated with lower rates of problem behaviors, about 23% lower for total, 22% lower for internalizing, and 21% lower for externalizing issues, compared to those meeting no recommendations. | 95 |
| 24-hour movement behaviours and self-rated health in Chinese adolescents: a questionnaire-based survey in Eastern China | Adolescents adhering to all three recommendations was associated with nearly a 12 times higher likelihood of reporting better self-rated health compared to meeting none. | 142 |
| 24-hour movement guidelines and suicidality among adolescents. | Meeting all three recommendations was linked to substantially lower odds of suicidal ideation (76% lower odds) and suicide attempts (92% lower odds). | 94 |
| 24-hour movement guidelines and weight status among preschool-aged children in Bangladesh: A community-level cross-sectional study | Adherence to all three guidelines was not associated with overweight or obesity. | 147 |
| Adherence to 24-h movement guidelines and cognitive difficulties in adolescents | Compared to those who did not meet any guidelines, adolescents meeting all three were 3.38 times more likely to report no cognitive difficulties. | 39 |
| Adherence to 24-h movement guidelines in Spanish schoolchildren and its association with insulin resistance: a cross-sectional study | Meeting one or none of the recommendations was associated with a 2.15 times higher risk of insulin resistance compared to meeting two or three recommendations, with an even higher risk observed in girls (2.80 times higher). | 149 |
| Adherence to 24-hour movement guidelines and their association with depressive symptoms in adolescents: Evidence from Bangladesh | Adolescents who met two or three recommendations had a 62% lower likelihood of experiencing depressive symptoms compared to those who did not meet any recommendations. | 161 |
| Adherence to 24-Hour Movement Guidelines in Relation to the Risk of Overweight and Obesity Among Children and Adolescents | Compared to those who did not adhere to any of the guidelines, participants who met all three recommendations had a 60% lower likelihood of developing overweight and obesity. | 160 |
| Adherence to 24-Hour Movement Recommendations and Health Indicators in Early Adolescence: Cross-Sectional and Longitudinal Associations in the Adolescent Brain Cognitive Development Study | Compared to those meeting no guidelines at T1, participants meeting all three had significantly better fluid, crystallized, and composite cognition scores, fewer behavioral problems, and lower adiposity. However, at T2 the cognitive benefits were no longer significant, although those meeting all three still showed lower adiposity and greater cortical gray matter volume compared to those meeting none. | 35 |
| Adherence to Combined Healthy Movement Behavior Guidelines among Adolescents: Effects on Cardiometabolic Health Markers | Adolescents who did not meet any guidelines were twice as likely to have higher cardiometabolic risk compared to those who met all three. | 17 |
| Adherence to the 24-hour movement guidelines and adiposity in a cohort of at risk youth: A longitudinal analysis | In childhood, compared to children meeting all three components, those meeting only two had a body mass index about 0.44 standard deviations higher, those meeting just one were about 0.85 standard deviations higher, and those meeting none were around 1.49 standard deviations higher; in early adolescence, children meeting only one component had a body mass index approximately 0.56 standard deviations higher than those meeting all three. | 33 |
| Are one-year changes in adherence to the 24-hour movement guidelines associated with depressive symptoms among youth? | Among females, meeting at least one more guideline than the previous year was associated with lower depressive symptoms, whereas for males, changes in guideline adherence were not linked to depressive symptoms after accounting for prior depression and follow-up factors. | 69 |
| Are one-year changes in adherence to the 24-hour movement guidelines associated with flourishing among Canadian youth? | Whether students increased their adherence from the previous year was not significantly associated with higher flourishing scores after controlling for depressive symptoms and other factors. | 72 |
| Association between 24‑hour movement behaviors and health‑related quality of life in children | Regression analysis indicated that each additional guideline met was associated with a 0.52-point higher PedsQL total score, with children meeting all three scoring 1.61 points higher than those meeting none. | 76 |
| Association Between 24-Hour Movement Guideline and Physical, Verbal, and Relational Forms of Bullying Among Chinese Adolescents | Compared with those meeting no recommendations, participants who met all three were 36% less likely to be physical victims, 66% less likely to be physical perpetrators, and 81% less likely to be bully-victims. Similarly, in terms of verbal bullying, those meeting all three were 33% less likely to be victims, and 65% less likely to be perpetrators or bully-victims. Moreover, a dose-response relationship was evident, with the likelihood of being involved in any bullying role decreasing as the number of recommendations met increased. | 177 |
| Association between 24-hour movement guidelines and physical fitness in children | Meeting all three guidelines was not linked to differences (compared to meeting none) in fitness measures such as grip strength (both absolute and relative to body weight), sit-ups in 30 seconds, sit-and-reach, or performance on the 20-meter shuttle run. | 90 |
| Association Between Combinations of 24-Hour Movement Behaviors and Depression Among Adolescents — Inner Mongolia Autonomous Region, China, 2019–2021 | In general, adherence to these guidelines was associated with lower rates of depression. Specifically, in 2019, adolescents who met none of the guidelines were 1.74 times more likely to report depression compared to those who met all three; this likelihood increased to 2.48 times in 2020 and 2.78 times in 2021. | 154 |
| Association between meeting the 24-h movement guidelines and psychosocial health in children: A cross-sectional study | Meeting all three guidelines was associated with fewer total difficulties, including reduced emotional symptoms, conduct problems, hyperactivity, and peer problems. Additionally, there was a dose‐response relationship, meaning that the more recommendations children met, the better their psychosocial health, particularly among boys. | 41 |
| Association between meeting the 24-hour movement guideline and anxiety status in Chinese school-aged adolescents | Not meeting any of the guidelines was associated with more than three times the likelihood of experiencing anxiety compared to those who met the guidelines. | 152 |
| Association between Physical Activity, Sedentary Behaviors, Sleep, Diet, and Adiposity among Children and Adolescents in China | Participants who met one or more guidelines had lower odds of overweight and obesity than those who met none, with the odds decreasing as the number of guidelines met increased. | 106 |
| Association between the 24-hour movement guidelines and executive function among Chinese children | In contrast, those who did not meet any recommendations showed significantly lower scores in Completed Categories and Shifting Efficiency, and higher scores in Non-Preservative Errors, indicating inferior global performance, reduced cognitive flexibility, and lower efficiency in rule discovery. | 38 |
| Association of Adolescent Bullying Victimization with Meeting 24‑hour Movement Behavior Recommendations: A Cross‑Sectional Study Using the Combined 2015–2019 Youth Risk Behavior Survey | Although the prevalence of meeting all three 24-h MB guidelines was reported, and the bullying-related outcomes of meeting individual guidelines were documented, the bullying-related outcomes of meeting all three guidelines together were not reported. | 124 |
| Associations between 24 hour movement behaviours and global cognition in US children: a cross-sectional observational study | Global cognition was positively associated with each additional recommendation met (an increase of 1.44 points), and those meeting all three had, on average, 3.89 points superior global cognition compared to those meeting none. | 36 |
| Associations between 24-h Movement Behavior and Internet Addiction in Adolescents: A Cross-Sectional Study | Children and adolescents who did not meet all three guidelines had an 8.44 times higher likelihood of Internet addiction compared to those who did meet these recommendations. | 127 |
| Associations between 24-h movement behaviors and self-rated health: a representative sample of school-aged children and adolescents in Okinawa, Japan | Meeting all three recommendations was strongly associated with a high prevalence of good health, with an odds ratio of 5.29 compared to those who met none. | 119 |
| Associations between combinations of 24‐h movement behaviors and physical fitness among Chinese adolescents: Sex and age disparities. | Specifically, meeting the MVPA, recreational screen time, and sleep guidelines together was associated with 1.81 times higher odds of achieving a high-level PFI compared to meeting none. | 162 |
| Associations between meeting 24-hour movement guidelines and mental well-being among Chinese adolescents in high-density cities | Adolescents who met all three guidelines had mental well-being scores that were 12.5 times higher compared to those who did not meet any guidelines. | 175 |
| Associations between meeting 24-hour movement guidelines and myopia among school-aged children: A cross-sectional study | Meeting all three guidelines was associated with a 60% lower risk of myopia compared to meeting none. Additionally, a greater number of guidelines met was linked to a reduced risk of myopia. | 153 |
| Associations between meeting combinations of 24-hour movement recommendations and dietary patterns of children: A 12-country study | Meeting all three guidelines was linked to the healthiest dietary patterns compared to meeting fewer recommendations. | 59 |
| Associations between meeting combinations of 24-h movement guidelines and health-related quality of life in children from 12 countries | Children who met all three recommendations reported a significantly higher overall Health-Related Quality of Life (HRQoL) T‐score (51.2) compared to those meeting none (49.6). | 54 |
| Associations between the Canadian 24 h movement guidelines and different types of bullying involvement among adolescents | Meeting all three recommendations was associated with notably lower risks of bullying: students meeting all three had about 68% lower odds of being a victim of school bullying, 75% lower odds of being both a bully and a victim, and 63% lower odds of being a victim of cyberbullying compared to those who did not meet any of the recommendations. | 92 |
| Associations of 24-Hour Movement Behavior with Depressive Symptoms and Anxiety in Children: Cross-Sectional Findings from a Chinese Sample | Compared to those meeting all three, participants who met none had 2.62 times higher odds of depressive symptoms and 2.32 times higher odds of anxiety. | 104 |
| Better health indicators of FitSpirit participants meeting 24-h movement guidelines for Canadian children and youth | Meeting all three recommendations was the strongest predictor of better perceived quality of life compared to meeting none. | 84 |
| Canadian 24-h Movement Guidelines, Life Stress, and Self-Esteem Among Adolescents | Meeting all three was associated with lower life stress (and higher self-esteem compared to meeting none. A clear dose-response gradient was observed, with greater numbers of recommendations met corresponding to lower life stress and higher self-esteem. | 128 |
| Combinations of physical activity, screen time and sleep, and their association with subjective wellbeing in children | Meeting all three was associated with 2 times better subjective wellbeing compared to meeting none. Moreover, a dose-response relationship was observed, with subjective wellbeing improving as the number of guidelines met increased. | 137 |
| Compliance With 24-Hour Movement Guidelines in Hong Kong Adolescents: Associations With Weight Status | There was no significant difference in BMI between those meeting all three guidelines and those not meeting them, for the overall sample, as well as for girls and boys separately. | 74 |
| Cross-sectional and longitudinal associations of adherence to the 24-hour movement guidelines with mental health problems among Chinese adolescents | Cross-sectionally, adolescents meeting all three recommendations were associated with significantly lower anxiety and depressive symptoms than those meeting none, approximately 2.8 units lower for anxiety and 3.4 units lower for depression. Six months later, those meeting all three guidelines were still associated with lower anxiety (about 1 unit lower) and depressive symptoms (about 1.4 units lower), and meeting all three was associated with approximately 61% lower odds of depressive symptoms. | 141 |
| Differential Associations Between Meeting 24-Hour Movement Guidelines With Mental Wellbeing and Mental Illness Among Chinese Adolescents | Compared with meeting no recommendations, meeting all three were significantly associated with better mental well-being and experienced lower severity of mental illness. Moreover, the relationship between the number of recommendations met and both mental well-being and mental illness indicators demonstrated a clear dose-response pattern. | 138 |
| Health associations with meeting new 24-h movement guidelines for Canadian children and youth | Meeting all three 24‐hour movement behaviours guidelines was linked with more favourable health outcomes, including lower Body Mass Index (BMI) z‐scores, waist circumference, blood pressure, insulin, triglycerides, and improved aerobic fitness, lipid profiles, better behavioral strengths and difficulties score, compared with meeting fewer recommendations. | 31 |
| Is adherence to the 24-hour movement guidelines associated with a reduced risk of adiposity among children and adolescents? | No significant differences in the odds of having a BMI z‐score >1 SD, excess fat mass, or excess visceral adipose tissue were observed between individuals meeting no guidelines and those meeting all three. | 73 |
| Is adherence to the 24-Hour Movement Guidelines associated with Mediterranean dietary patterns in adolescents? | Adolescents meeting all three guidelines showed higher adherence to the Mediterranean diet (β = 1.33) and were more likely to consume fruit and vegetables once a day, fish regularly, and eat cereal or grains for breakfast, while being less likely to consume commercially baked goods or sweets, compared to those not meeting the guidelines. | 109 |
| Is adherence to the Canadian 24-Hour Movement Behaviour Guidelines for Children and Youth associated with improved indicators of physical, mental, and social health? | Compared to those meeting fewer recommendations, children adhering to all three exhibited lower BMI and emotional problem scores, and higher life satisfaction. | 32 |
| Is meeting with the 24-h movement recommendations linked with suicidality? Results from a nationwide sample of 44,734 U.S. adolescents | Adolescents who met all three recommendations were 51% less likely to experience suicidal ideation, 49% less likely to engage in suicide planning, and 34% less likely to attempt suicide compared to those who did not meet all recommendations. Additionally, younger adolescents, female adolescents, and adolescents from minority racial backgrounds who adhered to the recommendations had a lower likelihood of suicide-related outcomes than those who did not. | 158 |
| Lifestyle Behavior and Mental Health in Early Adolescence | Children who met all three movement recommendations experienced 54% fewer mental health visits compared to those who met one or no recommendations. | 176 |
| Lifestyle Behaviours of Children and Adolescents During the First Two Waves of the COVID-19 Pandemic in Switzerland and Their Relation to Well-Being: An Observational Study | Those who met all three recommendations were more likely to report excellent health and higher life satisfaction compared to participants who did not meet any recommendations. | 116 |
| Longitudinal and cross-sectional associations of adherence to 24-hour movement guidelines with cardiometabolic risk | Meeting all three guidelines was linked to lower cardiometabolic risk factors, though these associations lost significance after adjusting for body fat. | 97 |
| Meeting 24 h Movement Guidelines and Health-Related Quality of Life in Youths during the COVID-19 Lockdown | Compared to those meeting all three, participants who met fewer guidelines had a higher likelihood of feeling worried, sad, or unhappy during the COVID-19 lockdown, and they reported lower overall health-related quality of life. | 111 |
| Meeting 24-h movement guidelines and associations with health related quality of life of Australian adolescents | Meeting all three recommendations was associated with an overall health-related quality of life score about 5 points higher, with physical and psychosocial scores around 5.2 and 4.8 points higher, respectively, compared to those meeting fewer recommendations. | 68 |
| Meeting 24-h movement guidelines: Prevalence, correlates, and associations with socioemotional behavior in Spanish minors | Compared to those meeting all three, participants meeting two, one, or none had progressively higher odds of socioemotional problems (with odds ratios of 1.42, 1.50, and 1.92, respectively). | 130 |
| Meeting 24-h movement guidelines: Prevalence, correlates, and the relationships with overweight and obesity among Chinese children and adolescents | Moreover, those who met the guidelines had lower odds of being overweight/obese. | 19 |
| Meeting 24-Hour Movement and Dietary Guidelines: Prevalence, Correlates and Association with Weight Status among Children and Adolescents: A National Cross-Sectional Study in China | Additionally, children who met only the sleep guideline had a higher risk of underweight compared to those meeting all three, while those who met only the physical activity or screen time guideline had a higher risk of overweight/obesity than children who met all three. | 131 |
| Meeting 24-Hour Movement Guidelines for Children and Youth and associations with psychological well-being among South Korean adolescents | Meeting all three guidelines was associated with a 1.29 times higher likelihood of being happy and a 1.46 times higher likelihood of not feeling stressed compared to meeting fewer guidelines. | 62 |
| Meeting the Canadian 24-Hour Movement Guidelines and physical-mental comorbidity among Chinese children and adolescents: Prevalence, associations, and the population impacts | Meeting more recommendations was associated with lower physical–mental comorbid risks, a dose–response relationship that was particularly strong among primary school students (with those meeting all three having 68% lower odds of comorbidity). At the population level, about 42.1% of comorbid cases were attributable to non-adherence, with potential prevention estimates of 30.8% for boys and 55.7% for girls if all children met all recommendations. | 156 |
| Movement and mental health: Behavioral correlates of anxiety and depression among children of 6–17 years old in the U.S. | Meeting all three guidelines was associated with a lower likelihood of anxiety and depression among adolescents, with those meeting no guidelines having more than twice the odds of anxiety compared to those meeting all three. | 66 |
| Movement Behaviors and Perceived Loneliness and Sadness within Alaskan Adolescents | Meeting all three guidelines was associated with notably lower odds of loneliness and, meeting three guidelines was linked to lower odds of prolonged sadness compared to meeting none. | 89 |
| Physical activity, screen time and sleep duration: Combined associations with psychosocial health among Canadian children and youth | Youth who met two/three of the recommendations were over three times more likely to experience positive psychosocial health compared to those who did not meet any guideline. | 40 |
| Proportion of children meeting recommendations for 24-hour movement guidelines and associations with adiposity in a 12-country study | Children meeting all three guidelines were approximately 72% less likely to be obese compared with those who met none. Combined adherence was also significantly associated with a lower Body Mass Index (BMI) z‐score. | 30 |
| Proportion of Chinese Children and Adolescents Meeting 24-Hour Movement Guidelines and Associations with Overweight and Obesity | Compared to those who met none of the guidelines, the odds of being overweight or obese were lower among those who met one (OR = 0.51), two (OR = 0.32), and all three (OR = 0.23) guidelines, with a significant decreasing trend in overweight/obesity rates. | 20 |
| Proportion of Japanese primary school children meeting recommendations for 24-h movement guidelines and associations with weight status | Children who met all three had lower odds of being overweight or obese compared to meeting none, after adjusting for age, gender, and socioeconomic status. | 87 |
| Prospective associations between adherence to 24-hour movement guidelines and mental well-being in Chinese adolescents | Meeting all three guidelines at baseline was not associated with beneficial changes in mental well-being over time compared to meeting none. | 148 |
| Relationship Between Meeting 24-Hour Movement Guidelines and Cardiometabolic Risk Factors in Children | Meeting all three guidelines was associated with lower odds of obesity compared to meeting fewer recommendation(s). | 57 |
| Relationship of 24-Hour Movement Behaviors with Weight Status and Body Composition in Chinese Primary School Children: A Cross-Sectional Study | Those who met all three guidelines had a 37% lower likelihood of being overweight or obese compared to those who did not meet them. | 120 |
| School-aged children’s movement behaviours and subjective health complaints in Japan: a cross-sectional study during COVID-19 pandemic-related school closures and after school reopenings | Those adhering to all three recommendations were more likely to be at low risk for symptoms related to physical and mental pain, fatigue, irritability, and lethargy compared to meeting none. | 165 |
| Sleep, sedentary behavior, and physical activity in Brazilian adolescents: Achievement recommendations and BMI associations through compositional data analysis | Although the prevalence of meeting all three 24-h MB guidelines was reported, the BMI outcomes associated with meeting all three guidelines were not reported. | 172 |
| The Association of Soft Drink Consumption and the 24-Hour Movement Guidelines with Suicidality among Adolescents of the United States | Adolescents who did not meet all the recommendations had significantly higher risks of suicidal ideation and suicide plans, about 1.7 times and 1.8 times higher, respectively, compared with those who met all the guidelines. | 125 |
| The Associations between Meeting 24-Hour Movement Guidelines (24-HMG) and Mental Health in Adolescents—Cross Sectional Evidence from China | Meeting all three guidelines was associated with a significantly lower risk of anxiety and depression in adolescents. | 18 |
| The associations between meeting 24-hour movement guidelines and adiposity in Asian Adolescents: The Asia-Fit Study | Moreover, after adjusting for region, age, gender, perceived health status, life satisfaction, perceived sleep quality, and dietary intake, a higher number of guidelines met was associated with a lower body fat percentage in Asian adolescents. | 34 |
| The Canadian 24-Hour Movement Guidelines and Psychological Distress among Adolescents | Only 4.7% of participants met all three recommendations, while 38.9% met none. Compared to those meeting no recommendations, meeting all three was associated with lower anxiety and depressive symptoms. | 93 |
| The Canadian 24‑hour movement guidelines and self‑rated physical and mental health among adolescents | Compared to those who met no recommendations, meeting all three was associated with significantly better self-rated physical health, over six times greater odds, and over three and a half times greater odds of positive self-rated mental health. | 102 |
| The Combinations of Physical Activity, Screen Time, and Sleep, and Their Associations with Self-Reported Physical Fitness in Children and Adolescents | Meeting all the guidelines was associated with significantly better general physical fitness, cardiorespiratory fitness, and muscular strength compared to those meeting none. | 112 |
| Trajectories of 24-h movement guidelines from middle adolescence to adulthood on depression and suicidal ideation: a 22-year follow-up study | Those who consistently met the guidelines had a substantially lower risk of depression symptoms and suicidal ideation compared to those who never met them. Additionally, individuals who did not meet the guidelines in adolescence but achieved them in adulthood also showed a lower risk of suicidal ideation. | 8 |
| Twenty-Four-Hour Movement Guidelines and Body Weight in Youth | Compared to those meeting all three, meeting zero guidelines was associated with a 1.85 times higher likelihood of overweight and a 4.25 times higher likelihood of obesity, with females showing even greater odds (nearly 5 times) compared to males (about 4 times) when meeting none. | 82 |
| Twenty‑four‑hour movement guidelines during adolescence and its association with obesity at adulthood: results from a nationally representative study | Adolescents who met all three had a lower risk of abdominal obesity in adulthood, they were 24% less likely to develop abdominal obesity than those who met none. | 10 |
| Twenty-four-hour movement guidelines during middle adolescence and their association with glucose outcomes and type 2 diabetes mellitus in adulthood | In Wave V, adolescents who met all three guidelines had a notably lower risk of developing type 2 diabetes compared to those who met none (prevalence ratio of 0.47). Additionally, with each extra guideline met, the risk of type 2 diabetes in adulthood was lower by 18% in Wave IV and 15% in Wave V. | 9 |
| Weight status, body composition, and diet quality of Spanish schoolchildren according to their level of adherence to the 24-hour movement guidelines | Low adherence (meeting 0 or 1 recommendation) associated with a higher percentage of overweight. Compared to those with medium (meeting 2 recommendations) or high adherence (meeting all 3 recommendations), the low adherence group also had lower fibre intake, lower intakes of vitamins B6, B12, C, selenium, and magnesium, and a higher intake of saturated fatty acids. | 70 |
| What is the role of adherence to 24-hour movement guidelines in relation to physical fitness components among adolescents? | Compared with those who met none, participants who met all three 24‐hour movement guidelines were about 3.3 times more likely to have high or very high cardiorespiratory fitness, nearly twice as likely to excel in the standing long jump, about twice as likely to demonstrate superior muscular fitness, and roughly twice as likely to have better overall physical fitness. | 143 |

# **Table 6.** Summary of findings of articles focused on academic performance outcomes associated with 24-hour movement behaviours guidelines adherence (listed in alphabetical order according to article title).

| Title | Summary of Findings | Manuscript Reference Number |
| --- | --- | --- |
| 24‑h movement behaviours in Spanish youth before and after 1‑year into the covid‑19 pandemic and its relationship to academic performance | Although the prevalence of meeting all three 24-h MB guidelines was reported, and the academic performance associated with individual behaviors was documented, the academic outcomes associated with meeting all three guidelines were not reported. | 110 |
| Adherence to 24-hour movement guidelines and academic performance in adolescents | Among middle school students, those meeting all three guidelines displayed better academic performance than those meeting none. | 23 |
| Association between meeting 24-h movement guidelines and academic performance in a sample of 67,281 Chinese children and adolescents | Meeting all three was associated with better academic performance in Chinese (1.56 times more likely), Math (1.51 times more likely), and English (1.73 times more likely) compared to meeting none. For primary school students, a higher number of guidelines met was linked to progressively better performance across all subjects. In junior middle school, better performance was observed with increased adherence, though not in a clear dose–response pattern. In high school students, however, meeting all three guidelines did not show a significant association with academic performance. | 145 |
| Associations between meeting 24-hour movement guidelines and academic achievement in Australian primary school-aged children | When assessed by self-report, meeting more guidelines was associated with higher numeracy achievement, with children meeting 1, 2, or 3 guidelines scoring 23, 34, and 40 points higher, respectively, than those meeting none, although no significant literacy differences were observed when using accelerometry. | 21 |
| Associations between meeting sleep, physical activity or screen time behaviour guidelines and academic performance in Australian school children | Children who met at least two of the guidelines showed better overall academic performance, including in maths and English. | 22 |
| Is adherence to 24-Hour Movement Guidelines associated with a higher academic achievement among adolescent males and females? | Overall, meeting all three guidelines was linked to higher academic achievement compared to meeting none. Among male adolescents, those meeting all three had significantly higher academic achievement than those meeting none. | 100 |
| Meeting 24-h Movement Guidelines is Related to Better Academic Achievement: Findings from the YRBS 2019 Cycle | Compared to those who met none, adolescents who met all three guidelines had 2.01 times higher odds of reporting first-class academic achievement. In sex-specific analyses, boys who met all guidelines had 2.05 times higher odds and girls had 2.26 times higher odds of first-class academic achievement. These significant associations were observed among adolescents in 9th to 10th grades, but not in higher grades. | 108 |
| Recommendations on screen time, sleep and physical activity: associations with academic achievement in Swiss adolescents | Meeting all three recommendations was associated with academic achievement scores that were, on average, 0.24 units higher compared to those who met none | 98 |

# **Table 7.** Summary of findings of articles focused on intervention focused on 24-hour movement behaviours guidelines (listed in alphabetical order according to article title).

| Title | Summary of Findings | Manuscript Reference Number |
| --- | --- | --- |
| Can High Schools Be an Effective Setting to Promote Healthy Lifestyles? Effects of a Multiple Behavior Change Intervention in Adolescents | Experimental school students exhibited significant improvements in meeting 24‐hour movement behaviours guidelines relative to control students and their own baseline measures. Furthermore, while the intervention was effective for both genders, the effect was notably larger in boys. | 178 |
| Exploring Parents' Message Receipt and Message Enactment of the World's First Integrated Movement Behaviour Guidelines for Children and Youth. | Parents who read the integrated guidelines were more likely to put the information into practice to help their child meet the recommendations, compared with those who read the segregated or control guidelines. In addition, the better the parents understood the message, the more likely they were to act on it. | 179 |
| Effects of a school-based intervention on physical activity, sleep duration, screen time, and diet in children | The prevalence of meeting all three behaviors together in this article was extracted from: Prevalence of meeting 24-Hour Movement Guidelines from pre-school to adolescence: A systematic review and meta-analysis including 387,437 participants and 23 countries (https://www.sciencedirect.com/science/article/pii/S2095254622000205). While the intervention had a significant effect on some individual behaviors, its impact on promoting all three behaviors together was not reported. | 126 |

Table 7. Summary of findings of articles focused on intervention focused on 24-hour movement behaviours guidelines (listed in alphabetical order according to article title).

| Study / programme | Setting & participants | Key components & theoretical basis | Delivery mode | Duration / dosage | Outcomes |
| --- | --- | --- | --- | --- | --- |
| Can High Schools Be an Effective Setting to Promote Healthy Lifestyles? Effects of a Multiple Behavior Change Intervention in Adolescents  Paths of the Pyrenees (Spain; adolescents, *n* ≈ 210) | Two secondary schools (One intervention, another control); whole-school community | Multicomponent MHBC ( multiple health behavior change) programme grounded in Social Ecological Model (SEM), Self-Determination Theory (SDT) & Theory of Planned Behavior (TPB); curricular (tutorial sessions, interdisciplinary project based learning, active school-break) + extracurricular (family workshops, health events); teacher need-support training | Teacher-led curricular actions; research-team facilitator co-ordinates; family involvement | One academic year; ≥ 12 tutorial sessions + ongoing school-wide actions | Experimental school students showed a significant improvement in meeting specific and general combinations of 24-hour movement guidelines |
| Exploring Parents' Message Receipt and Message Enactment of the World's First Integrated Movement Behaviour Guidelines for Children and Youth.  24-h guideline message framing (Canada; parents, *n* ≈ 1 300) | Online experiment with parents of 5–17 y children | Exposure to integrated vs. segregated vs. control guidelines; based on message-design theory & Health Action Process Approach | Survey platform (Qualtrics); guideline viewing, thought-listing, planning tasks | Single exposure; follow-up at 2 weeks | Parents’ initial receipt of a message is important for subsequent behaviour change in a youth movement behaviour context. |
| Effects of a school-based intervention on physical activity, sleep duration, screen time, and diet in children  Spanish CAS + SDT school intervention (primary school, *n* ≈ 120) | Two grade-4 classes; | Ten 1-h tutorial-action sessions; MBCTs (16 techniques) mapped to SDT & Competency-Autonomy-Relatedness; family engagement | Researcher-delivered sessions with teachers present & co-designing; indirect family & recess components | 2.5 months; 10 weekly sessions | Ten one-hour sessions of the school-based intervention conducted through the tutorial action plan seem effective in improving the proportion of active children. |
